# The Influence of Hepatitis C Virus Genetic Region on Phylogenetic Clustering Analysis

**DOI:** 10.1371/journal.pone.0131437

**Published:** 2015-07-20

**Authors:** François M. J. Lamoury, Brendan Jacka, Sofia Bartlett, Rowena A. Bull, Arthur Wong, Janaki Amin, Janke Schinkel, Art F. Poon, Gail V. Matthews, Jason Grebely, Gregory J. Dore, Tanya L. Applegate

**Affiliations:** 1 The Kirby Institute, University of New South Wales Australia, Sydney, Australia; 2 Inflammation and Infection Research Centre, School of Medical Sciences, University of New South Wales Australia, Sydney, Australia; 3 Academic Medical Centre, Department of Medical Microbiology, Section of Clinical Virology, Amsterdam, The Netherlands; 4 BC Centre for Excellence in HIV/AIDS, Vancouver, Canada; 5 Department of Medicine, University of British Columbia, Vancouver, Canada; 6 HIV/Immunology/Infectious Diseases Clinical Services Unit, St Vincent’s Hospital, Sydney, Australia; Institut Pasteur, FRANCE

## Abstract

Sequencing is important for understanding the molecular epidemiology and viral evolution of hepatitis C virus (HCV) infection. To date, there is little standardisation among sequencing protocols, in-part due to the high genetic diversity that is observed within HCV. This study aimed to develop a novel, practical sequencing protocol that covered both conserved and variable regions of the viral genome and assess the influence of each subregion, sequence concatenation and unrelated reference sequences on phylogenetic clustering analysis. The Core to the hypervariable region 1 (HVR1) of envelope-2 (E2) and non-structural-5B (NS5B) regions of the HCV genome were amplified and sequenced from participants from the Australian Trial in Acute Hepatitis C (ATAHC), a prospective study of the natural history and treatment of recent HCV infection. Phylogenetic trees were constructed using a general time-reversible substitution model and sensitivity analyses were completed for every subregion. Pairwise distance, genetic distance and bootstrap support were computed to assess the impact of HCV region on clustering results as measured by the identification and percentage of participants falling within all clusters, cluster size, average patristic distance, and bootstrap value. The Robinson-Foulds metrics was also used to compare phylogenetic trees among the different HCV regions. Our results demonstrated that the genomic region of HCV analysed influenced phylogenetic tree topology and clustering results. The HCV Core region alone was not suitable for clustering analysis; NS5B concatenation, the inclusion of reference sequences and removal of HVR1 all influenced clustering outcome. The Core-E2 region, which represented the highest genetic diversity and longest sequence length in this study, provides an ideal method for clustering analysis to address a range of molecular epidemiological questions.

## Introduction

Hepatitis C virus (HCV) is a member of the Flaviviridae family. The single positive RNA strand is 9.6 kilobases and encodes a polyprotein of about 3,000 amino acids. HCV is highly efficient at replication, with an estimated daily reproduction of 10^12^ new virions. The key enzyme for replication, the RNA-dependent RNA polymerase, which is encoded by NS5B, lacks proof-reading ability [1]. This results in the introduction of at least one mutation in the genome in each replicative cycle[2]. This error-prone replicase leads to the development of a diverse and continuously evolving population of viruses with variations in the genome moulded by host and virus selective pressures [3]. Seven HCV genotypes (1 to 7) with approximately 100 sub-types (1a, 1b, etc.) have been identified on the basis of molecular phylogenetic analyses of HCV sequences [4].

Sequencing is an important tool for understanding the molecular epidemiology and viral evolution of HCV infection. Given the diversity and secondary structure of HCV, it is often difficult to sequence longer regions of the genome in a time and cost effective manner. Consequently there is little standardisation among protocols with the HCV region sequenced and technology used influenced by the study aims [5]. While guidelines have been developed for genotyping and subtyping studies, no study has systematically evaluated the influence of selected regions on phylogenetic clustering to help inform guidelines for phylogenetic analyses. Clustering analyses of HCV genomes are generally performed using short sequences within the Core to E1/HVR1 or NS5B regions of HCV [6–9].

Phylogenetic analyses, including evolutionary and transmission studies, are known to improve through the use of longer fragments of HCV RNA [10] and fragments containing higher viral genome diversity [11]. The ideal HCV polymerase chain reaction (PCR) amplicon for phylogenetic analysis would (i) contain sufficient genetic information consisting of a range of genomic diversity to allow multiple downstream analyses, (ii) be large enough to encompass previously published methods to facilitate data sharing and cross-cohort analyses, and (iii) be practical and affordable.

While a full genome transcription and amplification method generating a single amplicon is now available [12], it is not yet known which of the smaller subregions will provide robust phylogenetic clustering results. This study aimed to develop a Core-E2 HCV sequencing protocol able to amplify multiple genotypes and contain sufficiently diverse genetic information suitable for a range of molecular epidemiological research questions. Our objective was to systematically analyse the influence of HCV subregions and concatenation of sequences on inferred transmission clusters in the Australian Trial in Acute Hepatitis C (ATAHC) study. Using a novel HCV sequencing protocol, this study demonstrates that the selection of HCV regions can affect the identification of clusters and offers a practical research tool to improve reliability of phylogenetic analysis.

## Materials and Methods

### Study population and design

ATAHC was a multicentre, prospective cohort study of the natural history and treatment of recent HCV infection, as previously described [13, 14]. Recruitment of HIV infected and HIV uninfected participants was from June 2004 through November 2007. Recent infection with either acute or early chronic HCV infection with the following eligibility criteria:

First positive anti-HCV antibody within 6 months of enrolment; and either
  - Acute clinical hepatitis C infection, defined as symptomatic seroconversion illness or alanine aminotransferase (ALT) level greater than 10 times the upper limit of normal (>400 IU/mL) with exclusion of other causes of acute hepatitis, at most 12 months before the initial positive anti-HCV antibody; or
  - Asymptomatic hepatitis C infection with seroconversion, defined by a negative anti-HCV antibody in the two years prior to the initial positive anti-HCV antibody.

The first available viraemic time point following acute HCV detection from all participants was included in this study. When enrolled in the ATAHC study, all participants provided written informed consent for future hepatitis C related research samples, which was approved by St Vincent’s Hospital, Sydney Human Research Ethics Committee (primary study committee) as well as through local ethics committees at all study sites. The ATAHC study was registered with clinicaltrials.gov registry (NCT00192569). This study called “Transmission, epidemiology and natural history of acute hepatitis C virus infection” has been approved by the St Vincent's Hospital Human Research Ethics Committee (HREC ref#LNR/12/SVH/223) for the sequencing and phylogenetic analysis of ATAHC samples.

### Detection and quantification of HCV RNA

Qualitative and quantitative HCV RNA testing was performed using the Versant TMA assay (Bayer, Australia; <10 IU/ml) and the Versant HCV RNA 3.0 (Bayer, Australia; <615 IU/ml), respectively.

### HCV RNA sequencing

#### Viral RNA extraction and reverse transcription

HCV RNAs were extracted from 140 μL of plasma of patient samples using QIAamp viral extraction mini kit (Qiagen) according to manufacturers’ instructions and eluted in 80 μl buffer. Reverse transcription was performed with random hexamers using the superscript VILO cDNA synthesizer kit (Life Technologies), containing 5 μL RNA, 1 μL Superscript Enzyme Mix, 2 μL VILO reaction Mix and 2 μL of PCR additive PolyMate (Bioline, UK). Reactions were heated on a thermocycler (Verity, Life Technologies) for 25°C for 10 minutes, 60°C for one hour and 85°C for 5 minutes.

#### Generation and sequencing of Core-E2 and NS5B amplicon

DNA was generated using SuperScript VILO cDNA Synthesis Kit (Life Technologies, Carlsbad, CA) with random hexamers. A 1,514 bp fragment of the HCV genome covering Core, Envelope-1, hypervariable region-1, and beginning of Envelope-2 (E2) was amplified using a method described in S1 Fig and S1 Table. NS5B (388bp) was amplified by a single round PCR as previously described [15] with some modifications to reaction conditions described in S1 File. Purified amplicons were sequenced using the Sanger method, described in S2 Fig. The Core-E2_NS5B sequences are available in Genbank with accession number KR855579 to KR855628.

#### Genotyping

The genotype was determined for all subjects for both Core-E2 and NS5B sequences using the Oxford HCV Automated Subtyping Tool (http://www.bioafrica.net/rega-genotype/html/subtypinghcv.html) [16].

### Phylogenetic and clustering analysis

**Fig 1:**
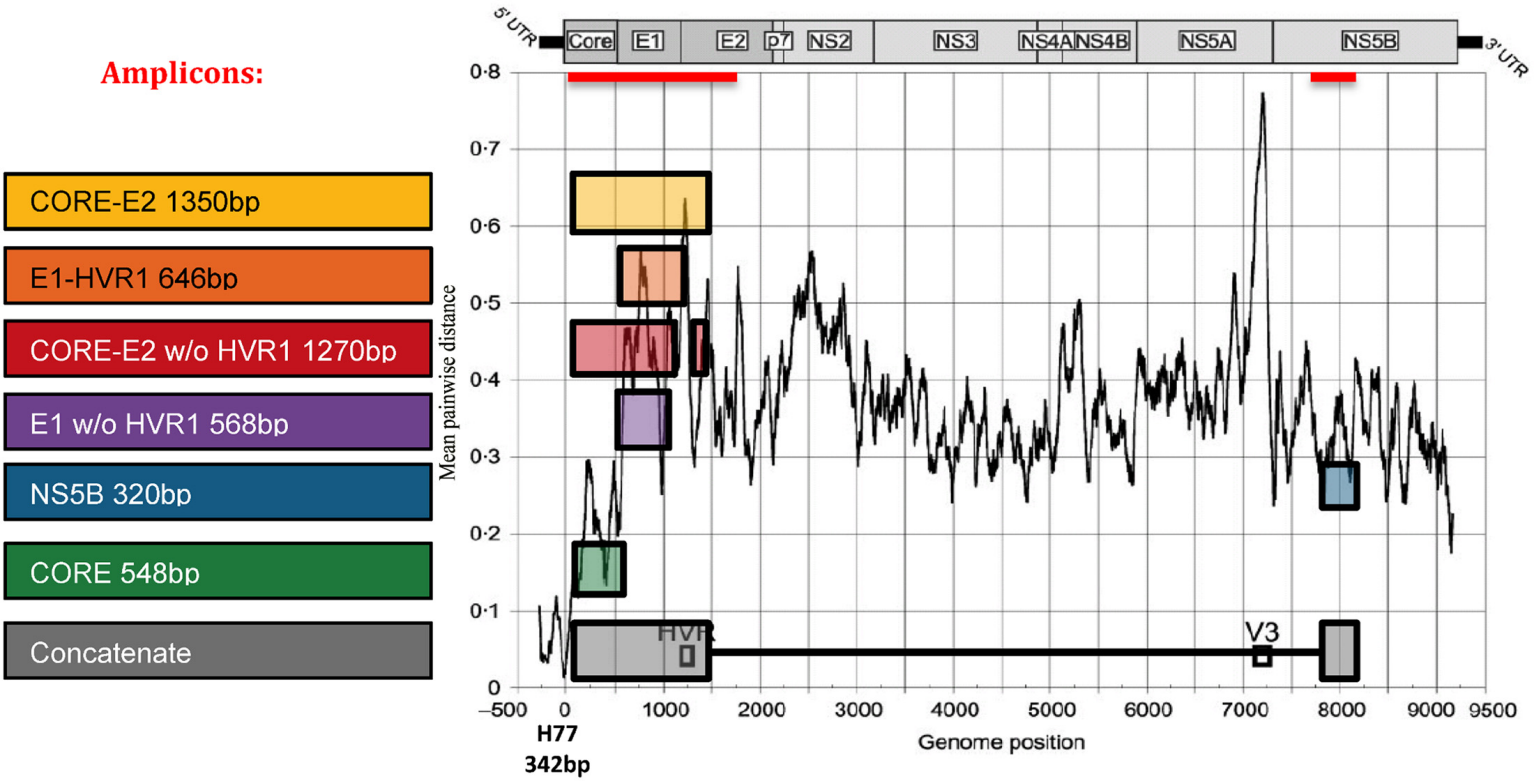
HCV amplicons and regions used for clustering analysis. The genetic variability between each region was estimated using the diagrammatic representation of the HCV genome from [50] as shown in mean pairwise distance from Fig 1 (black line). The black line represents the genetic diversity across the length of HCV RNA [50]. The red lines represent the amplicons generated with the in-house Core-E2 protocol (1534bp) and the NS5B method published by Murphy *et al*. (360bp) [15]. The colour rectangles show the location of the sequences used for clustering analysis: the full Core-E2 amplicon; same sequence trimmed to E1 with partial HVR1; with HVR1 removed; E1 alone, without HVR1; NS5B; Core. Every sequence from the 5’ region was analysed alone and also concatenated to NS5B. Full length sequences from naïve GT1a patients available from LANL were trimmed to identical regions to be used as reference sequences.

Eleven scenarios, generated from seven subregions from within Core-E2 and NS5B regions, analysed either alone or concatenated with NS5B, were compared to assess their influence on phylogenetic clustering (Fig 1). Sequences were aligned (ClustalW) and, employing a general time-reversible (GTR) substitution model, phylogenetic trees were inferred using maximum-likelihood analysis (RAxML) [17]. Trees were visualised with FigTree (http://tree.bio.ed.ac.uk/software/figtree/ designed by A. Rambaut, version 1.3.1 –December 2009), using a bootstrap test with 1000 replicates and 90% cut-off for defining clusters. The influence of the inclusion of 126 unrelated reference sequences from the LANL database during analysis was also assessed.

**Fig 2:**
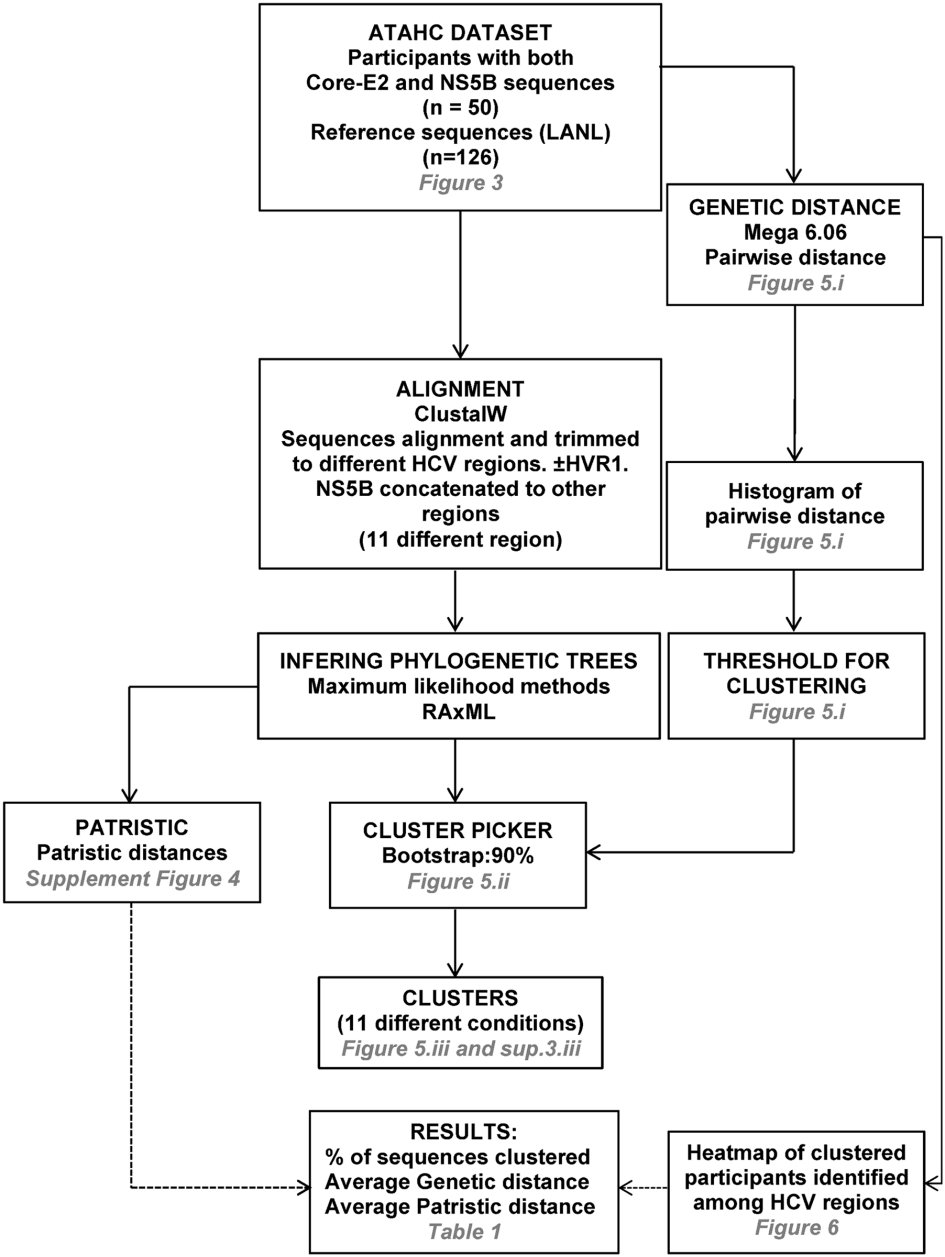
Flow chart describing the phylogenetic analysis. Sequences from 50 GT1a ATAHC participants and 126 reference sequences from the Los Alamos National Laboratory were analysed for several parameters with Mega 6.06 (pairwise distances, distribution and heatmap), ClustalW (sequence alignment), RAxML (inferring tree with Maximum Likelihood), Cluster Picker (number of clusters, average cluster size, average genetic distance and participants ID in a cluster) and PATRISTIC (patristic distance).

A sensitivity analysis for each region (plus or minus reference sequences) was completed to assess the impact of difference genetic variability on the percentage of cluster sequences and the average cluster size, as determined by Cluster Picker [18] using a fixed bootstrap support of 90% (See Fig 2 for description of process for phylogenetic analysis). This Java-based program identifies clusters of sequences in a phylogenetic tree based on support for the node (bootstrap or posterior probability) and the maximum pairwise genetic distance within the cluster. A second sensitivity analysis for each region (plus or minus reference sequences) was completed to assess the impact of varying the genetic variability on the average cluster patristic distance and the average bootstrap value as determined by PATRISTIC [19], a Java-based program that calculates patristic distances from large trees. Patristic distances are the sum of the length of the branches that connect two nodes in a phylogenetic tree, where those nodes are typically terminal nodes representing extant taxa. This is an inferred distance based on tree topology rather than the crude genetic distance directly computed from a pairwise comparison of two sequences [20].

A genetic distance “clustering threshold” was estimated for each region using the distribution of genetic distance for both ATAHC participants and LANL reference sequences. For every two sequences, the distance is a single value based on the fraction of positions in which the two sequences differ and calculated with Mega version 6.0 [21]. Results were represented as histogram with intervals based on bins of 0.005 distances (1225 and 7875 distance values for ATAHC and LANL respectively). The thresholds for clustering were estimated by determining the point of overlap/uncertainty region between the two curves of most-closely related (among ATAHC sequences) and distantly related (both ATAHC and LANL sequences) sequences for each region. Our hypothesis was that local Australian ATAHC sequences with genetic distances below this threshold would be identified as pairs or clusters, while most LANL sequences from worldwide origin would be likely to have genetic distances above this threshold.

The clustering threshold (genetic distance) was used for both sensitivity analyses to compare the percentage of sequences that clustered, the average cluster size (as determined by Cluster Picker), the average patristic distance of each cluster (PATRISTIC) and the average bootstrap value of each region. A sensitivity analysis for regions Core-E2, Core-E2_NS5B and NS5B was completed to assess the impact of varying the bootstrap support threshold, 70 to 98%, using Cluster Picker, first using region specific genetic clustering threshold and then set at 8% genetic threshold for all regions. The 8% genetic distance threshold was selected to include the largest number of clustered sequences to restrict the impact on clustering by varying bootstrap support threshold alone. Pairwise distance was then analysed to assess how subregions affected individual sequences falling within a cluster. Sequences with a pairwise distance below the genetic threshold (as defined in previous paragraph) were identified as part of a cluster represented as heatmap. The colours represent the number of regions which were consistently identified each sequence in a cluster. A phylogram based on the Core-E2 analysis highlights sequences that are included/excluded of cluster depending of the region selected.

The impact of HCV region on phylogenetic tree topology was also assessed using a final method, weighted Robinson-Foulds (RF) metric tree distance [22, 23] measured by RAxML. It is a symmetric difference metric of unrooted phylogenetic trees. The reference tree used for this measurement was Core-E2 concatenated with NS5B, as the longest available sequence to provide the most objective baseline. The first computed weighted RF value was used and evaluated with the mean genetic distance, calculated by Mega 6, and the length of sequences from the 50 GT1a sequences of ATAHC. The mean genetic distance for all 50 GT1a ATAHC sequences, among all regions, were compared using Mega 6 analysis to compute overall mean distance, using nucleotide substitution models and p-distance method with partial deletion (95% site coverage cut-off) as shown in Table 1 (Mean genetic distance) [24].

**Table 1:** Clustering characteristics of different HCV subregions, derived from the Core-HVR1 amplicon and NS5B. Characteristics of each subregion were determined after sequence alignment and gaps deletion, including sequence length, H77 sequence location within HCV, genetic diversity calculated from P. Simmonds (as per Fig 1).;clustering threshold estimated from the genetic distance distribution; percentage of sequences clustered at threshold; average cluster size; average patristic distance of identified clusters; average bootstrap values of identified clusters; percentage of sequences clustered using pairwise distance threshold and no bootstrap support.

HCV Region:	Sequence length*	H77 sequence location (length)	Mean genetic distance^$^	Genetic distance threshold^#^	Percentage of sequence clustered^&^		Average cluster size	Average patristic distance of identified clusters		Average bootstrap valuesof identified clusters		Percentage of sequence clustered using pairwise distance threshold^@^
					ATAHC	ATAHC + Ref^‡^	ATAHC	ATAHC	ATAHC + Ref^‡^	ATAHC	ATAHC + Ref^‡^	ATAHC
CORE-E2	1350	367_1730 (1364)	0.082	0.045	32	33	2.7	0.031	0.088	99.8	99.8	40
E1-HVR1	646	914_1571 (658)	0.112	0.060	28	28	2.3	0.045	0.084	99.6	99.6	40
CORE-E2 w/o HVR1	1270	367_1730 (1364)	0.068	0.030	26	26	2.6	0.017	0.020	98.3	98.8	36
E1	568	914_1490 (577)	0.086	0.035	23	26	2.3	0.017	0.017	98.6	98.4	36
NS5B	320	8286_8616 (331)	0.054	0.015	15	6	2.0	0.007	0.003	96.3	100	20
CORE	548	367_914 (548)	0.033	Not suitable	-	-	-	-	-	-	-	-
CORE-E2_NS5B	1670	367_1730 8286_8616	0.076	0.050	40	40	2.8	0.031	0.098	99.8	100	36
E1-HVR1_NS5B	966	914_1571 8286_8616	0.092	0.055	33	31	2.7	0.044	0.103	98.3	99.3	32
CORE-E2 w/o HVR1_NS5B	1590	367_1730 8286_8616	0.065	0.030	18	24	3.0	0.014	0.018	99.3	96.7	28
E1 _NS5B	888	914_1490 8286_8616	0.074	0.035	18	18	3.0	0.012	0.014	99.1	99.3	24
CORE_NS5B	868	367_914 8286_8616	0.041	0.015	16	16	2.0	0.012	0.022	98.3	98.7	20

*: Sequence length after alignment and gaps deletion;

^$^:Genetic distance calculated with ATAHC sequences;

^#^:Genetic distance threshold estimated from ATAHC pairwise distance distribution;

^&^:Percentage of sequence clustered using region genetic distance threshold using cluster picker and bootstrap at 90%;

^@^: Percentage of sequence clustered using pairwise distance threshold (without any bootstrap threshold);

^‡^: ATAHC sequences with LANL reference sequences.

## Results

### Participant characteristics

**Fig 3:**
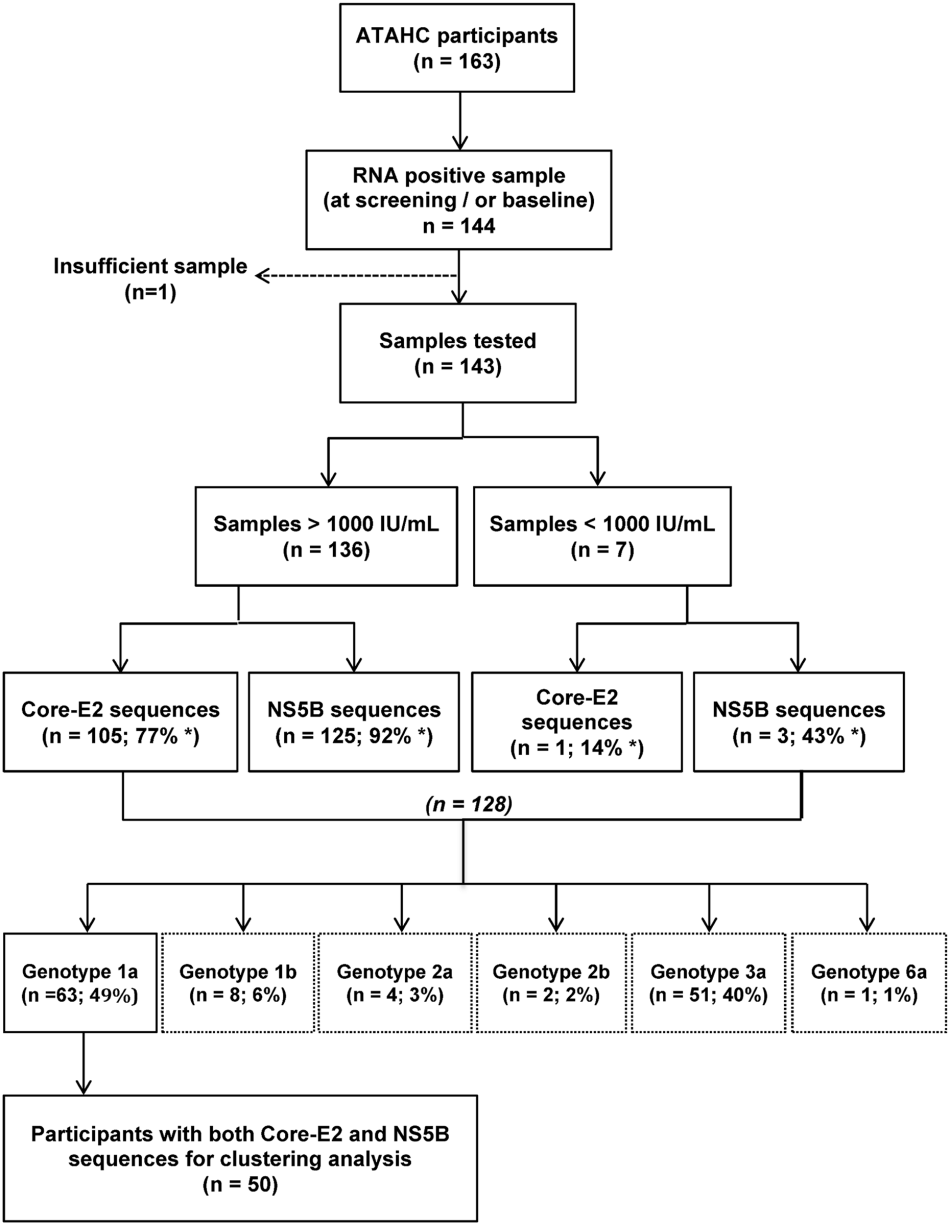
Flow chart describing the selection of ATAHC participants for inclusion in this analysis. Among the ATAHC participants (n = 163), 143 samples were tested and sequences were generated for Core-E2 and NS5B regions. 50 participants with both Core-E2 and NS5B sequences were used for clustering analysis.

Overall, samples with detectable HCV RNA were available from 143 of 163 participants enrolled in the ATAHC study. In total, 106 Core-E2 and 128 NS5B sequences were generated giving a success rate of 74% and 90% respectively. The genotype distribution among these 128 samples is 49% GT1a, 40% GT3a, 6% GT1b, 3% GT2a, 2% GT2b and 1% GT6k. As a greater number of related sequences were found within GT1a participants, only GT1a participants for whom both Core-E2 and NS5B amplicons were available were included in the phylogenetic analysis (n = 50) (Fig 3).

### Mean genetic distance among HCV regions

The clustering characteristics of different HCV subregions, derived from Core-E2 and NS5B are shown in Table 1.

**Fig 4:**
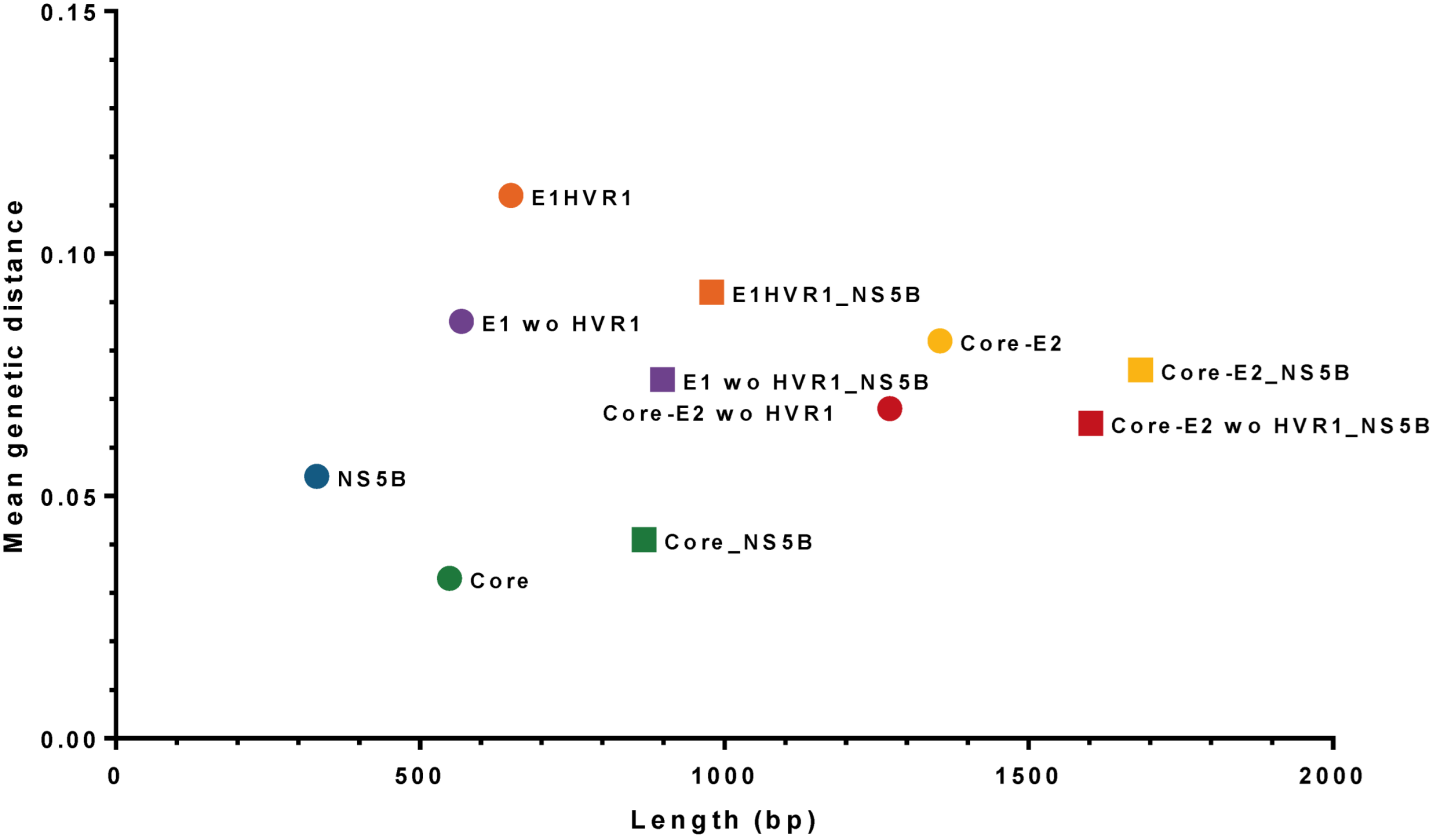
Mean genetic distance versus length of HCV regions. Relationship between mean genetic distance and length of HCV regions used in this project (squares for concatenated regions; circles for single regions). Regions with high mean genetic distance and longer size are preferable for phylogenetic analysis.

The highest and lowest mean genetic distances were observed in E1-HVR1 and Core regions respectively (Table 1, S5 Fig). Core-E2 demonstrated a lower mean genetic distance as compared to E1-HVR1, given it includes a large part of the Core region. Core-E2 provided the greatest genetic distance and longest sequence length for any single region (except when concatenated with NS5B) (Fig 4). The removal of HVR1 decreased the mean genetic distance for the regions involved (Figs 4 and S4). Concatenating NS5B to the structural regions decreased the mean genetic distance for all regions except Core.

### The impact of HCV region on defining clusters

#### HCV region influenced the genetic distance threshold for clustering

**Fig 5:**
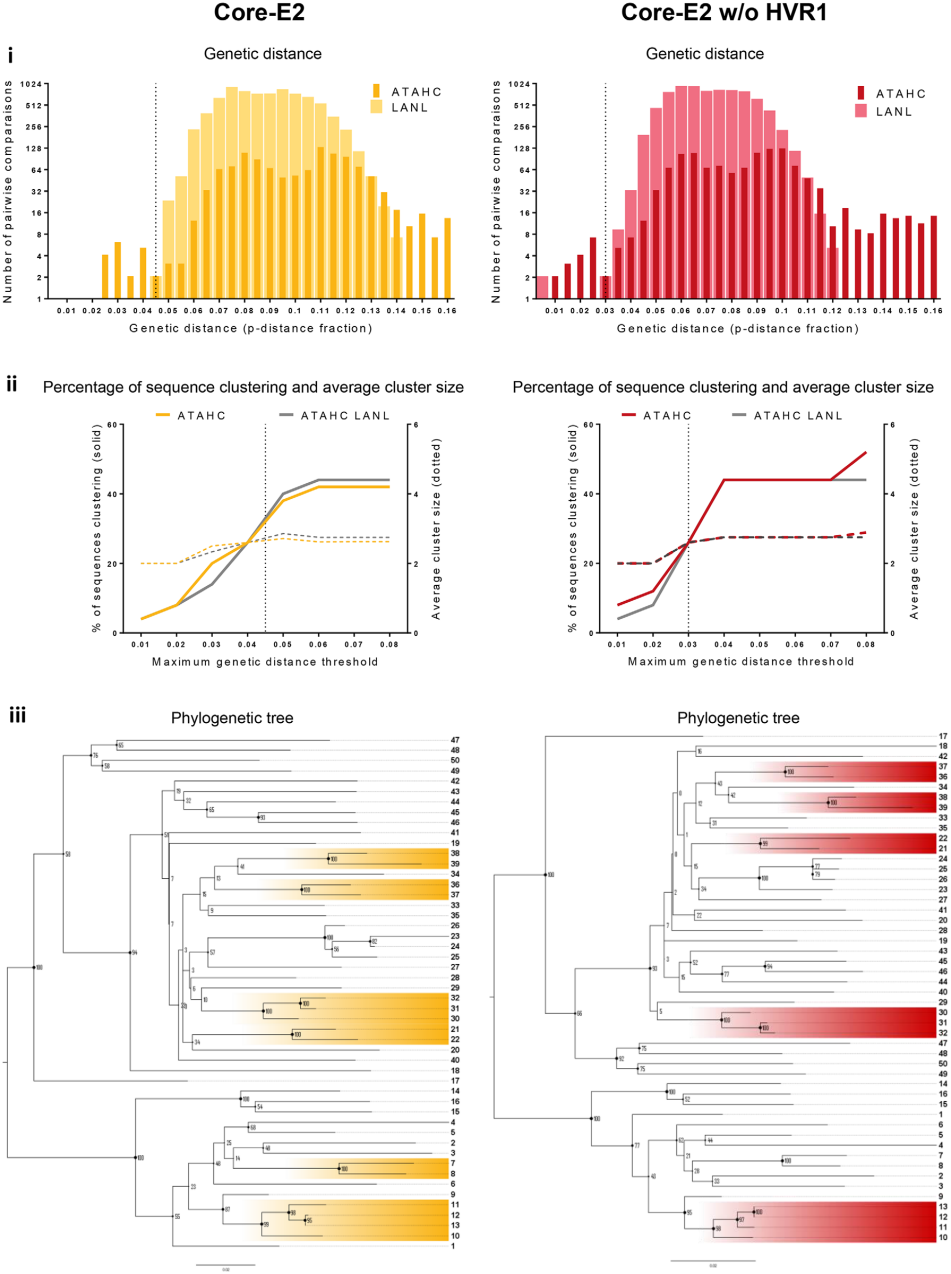
Clustering results among the 50 GT1a ATAHC sequences: genetic distance, percentage of sequences, tree, patristic distance and bootstrap values. *Panel i*: The genetic distance distribution is shown for both ATAHC sequences (dark colour) and Los Alamos HCV database reference sequences (clear colour). The vertical dotted lines represent the thresholds for clustering, which were estimated by determining the point of overlap/uncertainty region between the two curves of most-closely related (ATAHC sequences) and distantly related (both ATAHC and LANL sequences) for each HCV region. *Panel ii* shows the ATAHC clustering patterns using Cluster Picker with bootstrap support threshold fixed at 90% and maximum genetic distance threshold varied between 0.01 and 0.08 (colour lines: ATAHC sequences; grey lines: LANL reference sequences). Plain lines represent the percentage of clustered sequences; dot lines correspond to average cluster size. The vertical dot line indicates the clustering threshold (as per panel i) used to determine the percentage of clustered sequences and average cluster size (Table 1). *Panel iii* shows the phylloclade with participants highlighted when defined as part of a cluster with the clustering threshold (panel i) and bootstrap support above 90% criteria (Cluster Picker).

**Fig 6:**
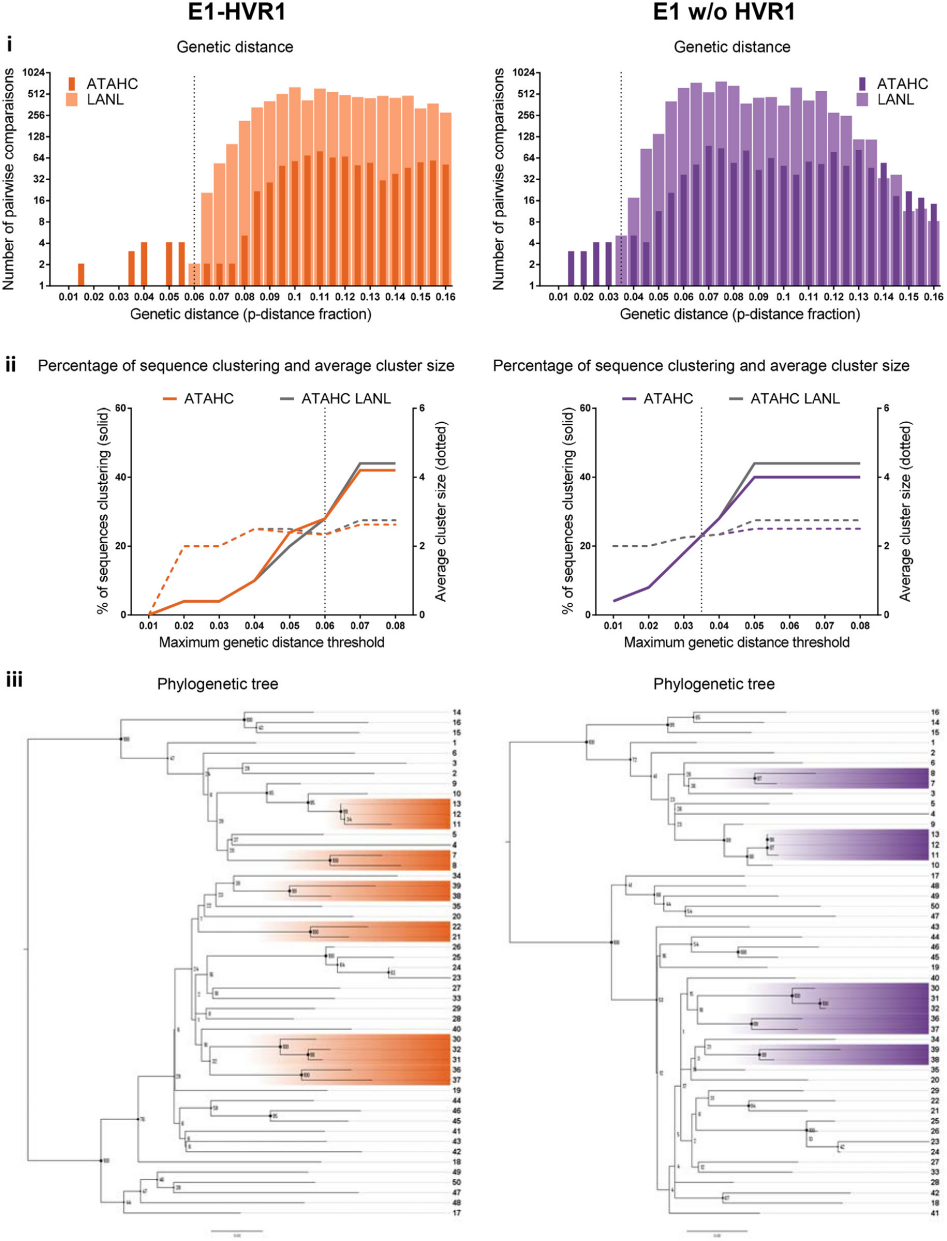
Clustering results among the 50 GT1a ATAHC sequences: genetic distance, percentage of sequences, tree, patristic distance and bootstrap values. *Panel i*: The genetic distance distribution is shown for both ATAHC sequences (dark colour) and Los Alamos HCV database reference sequences (clear colour). The vertical dotted lines represent the thresholds for clustering, which were estimated by determining the point of overlap/uncertainty region between the two curves of most-closely related (ATAHC sequences) and distantly related (both ATAHC and LANL sequences) for each HCV region. *Panel ii* shows the ATAHC clustering patterns using Cluster Picker with bootstrap support threshold fixed at 90% and maximum genetic distance threshold varied between 0.01 and 0.08 (colour lines: ATAHC sequences; grey lines: LANL reference sequences). Plain lines represent the percentage of clustered sequences; dot lines correspond to average cluster size. The vertical dot line indicates the clustering threshold (as per panel i) used to determine the percentage of clustered sequences and average cluster size (Table 1). *Panel iii* shows the phylloclade with participants highlighted when defined as part of a cluster with the clustering threshold (panel i) and bootstrap support above 90% criteria (Cluster Picker).

**Fig 7:**
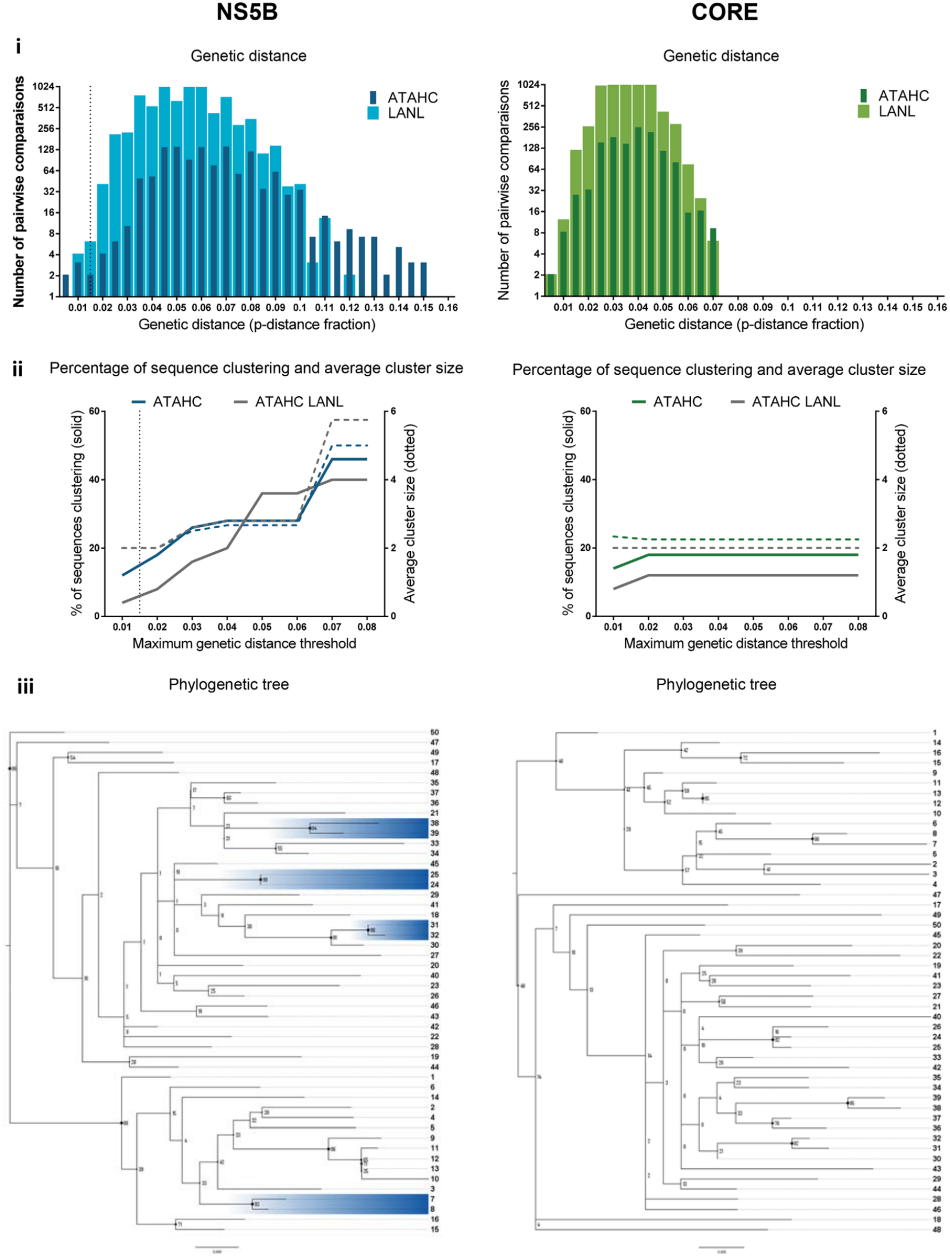
Clustering results among the 50 GT1a ATAHC sequences: genetic distance, percentage of sequences, tree, patristic distance and bootstrap values. *Panel i*: The genetic distance distribution is shown for both ATAHC sequences (dark colour) and Los Alamos HCV database reference sequences (clear colour). The vertical dotted lines represent the thresholds for clustering, which were estimated by determining the point of overlap/uncertainty region between the two curves of most-closely related (ATAHC sequences) and distantly related (both ATAHC and LANL sequences) for each HCV region. *Panel ii* shows the ATAHC clustering patterns using Cluster Picker with bootstrap support threshold fixed at 90% and maximum genetic distance threshold varied between 0.01 and 0.08 (colour lines: ATAHC sequences; grey lines: LANL reference sequences). Plain lines represent the percentage of clustered sequences; dot lines correspond to average cluster size. The vertical dot line indicates the clustering threshold (as per panel i) used to determine the percentage of clustered sequences and average cluster size (Table 1). *Panel iii* shows the phylloclade with participants highlighted when defined as part of a cluster with the clustering threshold (panel i) and bootstrap support above 90% criteria (Cluster Picker).

The distribution of genetic distance for ATAHC and LANL sequences for each region were determined (Figs 5–7, panel i; see also S3–S5 Figs, panel i). A threshold for clustering (represented by vertical dotted line in panel i and ii in Figs 5–7) was estimated from this distribution for ten out of the eleven regions by differentiating most-closely and distantly related ATAHC sequences, as we assumed these two sequence groups are distinctively visualised and the threshold identified by the point of overlap/uncertainty region between the two curves. Pairwise distance distribution of the LANL sequences was similar to distantly related ATAHC sequences distribution (Figs 5–7, panel i).

The Core-E2 region clustering threshold of 0.045 genetic distance decreased to 0.03 once HVR1 was removed (Fig 5, panel i, Core-E2 and Core-E2 w/o HVR1). The E1-HVR1 region demonstrated a wider distribution and the highest clustering threshold (0.06 genetic distance; Fig 6, panel i). A clustering threshold was unable to be estimated for the Core region due to the narrow low distribution of the genetic distances of this sequence, although a threshold of 0.015 was possible once concatenated to NS5B (Fig 7, panel i, NS5B and S3–S5 Figs, panel i). Concatenation of HCV regions with NS5B only moderately affected the clustering threshold in all other regions. Overall, removing HVR1 shifted the pairwise distribution curves to the left, decrease of genetic distance, as seen for example for Core-E2 and E1-HVR1 (Fig 5, panel i).

#### HCV region influenced the clustering pattern

Clustering patterns were evaluated for every region using Cluster Picker with bootstrap support threshold fixed at 90% and maximum genetic distance threshold varied between 0.01 and 0.08 (Figs 5–7 and S3–S5, panel ii). The dotted vertical lines represent the clustering threshold as previously described (panel i Figs 5–7). Larger regions such as datasets Core-E2, Core-E2 w/o HVR1, E1-HVR1 and E1 w/o HVR1 all showed a similar pattern, demonstrating a regular increase in the percentage of sequences clustering until a plateau of 40–44% was reached between 0.04 and 0.07 genetic distance (panel ii Figs 5–7). The maximum genetic distance at which this clustering plateau was reached was different for each region.

The clustering threshold for each region estimated by the genetic distance distribution defined the percentage of ATAHC sequences clustered from the Cluster Picker output (Figs 5–7 panel ii and S3–S5 Figs, panel ii, vertical dotted lines and Table 1). Concatenation with NS5B increased the percentage of sequences clustered for most regions, with the exception of Core-E2 without HVR1. Adding LANL references to the ATAHC sequences had limited effect on the percentage on sequences clustered, with the exception of NS5B, which showed a decrease from 15% to 6% sequences clustered (Fig 7, panel ii and Table 1). Overall, HVR1 influenced the clustering pattern, where the plateau was reached more rapidly when HVR1 was removed and the percentage of sequences clustered reduced. The removal of HVR1 from Core-E2 decreased clustering from 32% to 26% and E1-HVR1 from 28% to 23% (Figs 5 and 6, panel ii and Table 1).

### HCV region influenced tree topology

Phylograms of the 50 GT1a ATHAC participants were generated for each region and midpoint rooted and bootstrap values for every node (Figs 5–7 and S3, S4 and S6, panel iii). The overall tree topology was similar for all regions with two main nodes, the first node contained 16 participants (participant from 1 to 16) and the second 34 (participant 17 to 50). Nevertheless, some minor topology variations amongst the outer nodes occurred with or without HVR1 (for example: participants 47, 48, 49, 50 and 17 between regions Core-E2, Core-E2 w/o HVR1 and E1-HVR1, E1 w/o HVR1; Figs 5 and 6 panel iii). With NS5B alone (Fig 7, panel iii), the first internal nodes demonstrated lower bootstrap support values suggesting it was difficult to assign relationships for some sequences in this tree, likely a result of higher sequence homology within this region. Nevertheless, those sequences involved in variation of tree topology were never classified as part of a cluster in any regions.

#### HCV region influenced the patristic distance of clustered sequences

Sensitivity analysis demonstrated HVR1 influenced the patristic distances of clusters and this influence was accentuated when reference sequences were included in the analysis. Patristic distances were increased two to three times for regions containing HVR1 when reference sequences were added to the analysis (S6 and S7 Figs and Table 1). However, for the regions without HVR1, the patristic distance was unaffected by the addition of reference sequences.

The average bootstrap values among identified clusters were high for all regions. NS5B alone was the lowest with 96.3%, which increased to 100% when references were added to the analysis (S6 and S7 Figs and Table 1). All other regions ranged between 98.3 to 99.8%. Bootstrap values slightly decreased when HVR1 was removed but all values remained above 98.3%.

### The impact of varying bootstrap values on clustering

#### Bootstrap influences the inclusion of sequences in clusters

**Fig 8:**
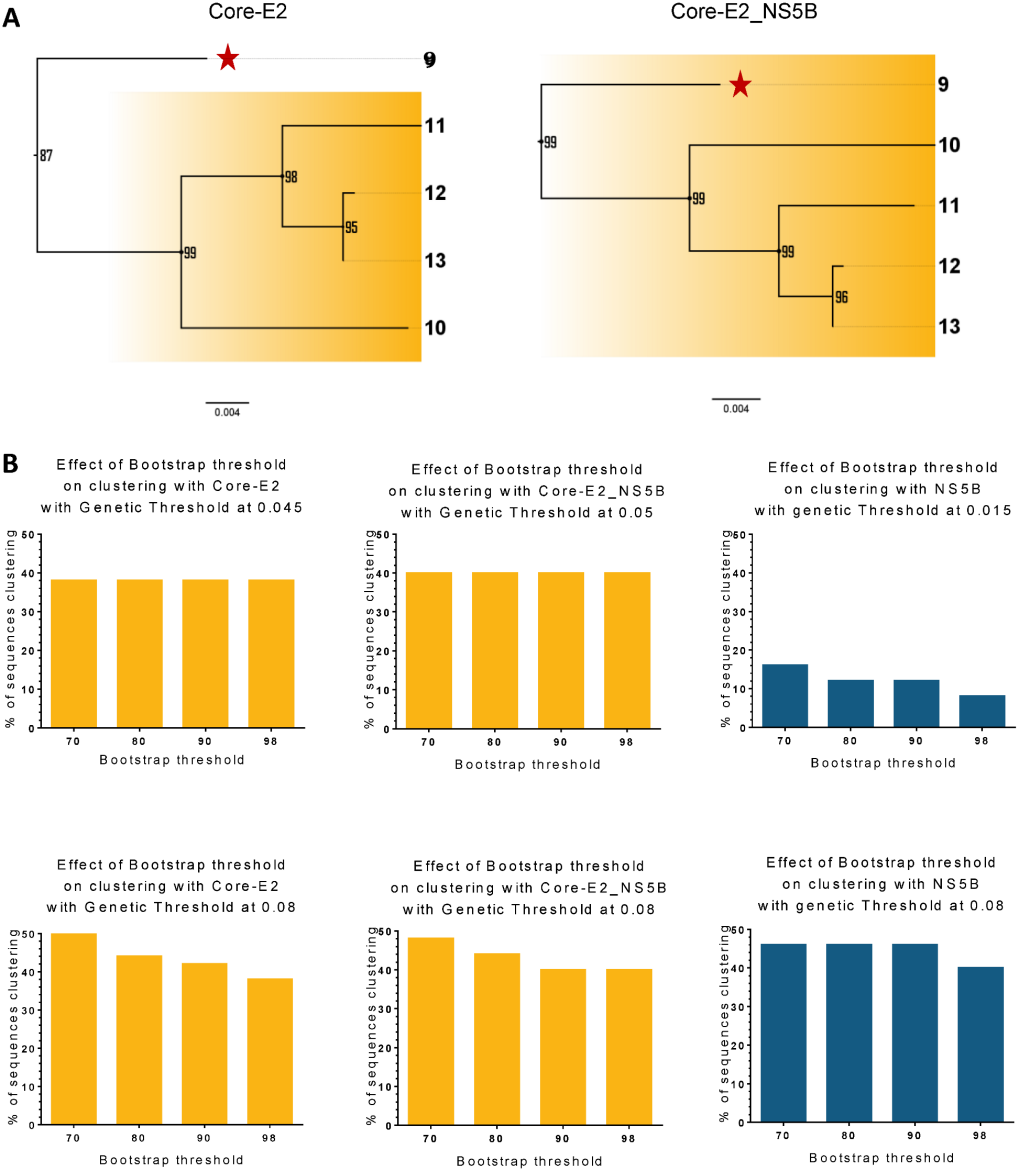
(A) Example of the influence of bootstrap value in cluster 1 identification using Core-E2 (scenario A) and Core-E2 concatenated with NS5B (scenario AE). (B) Bootstrap threshold can affect clustering depending of the region considered (Core-E2, Core-E2_NS5B or NS5B) (varying genetic threshold (top panel) or constant genetic threshold (bottom panel)).

**Fig 9:**
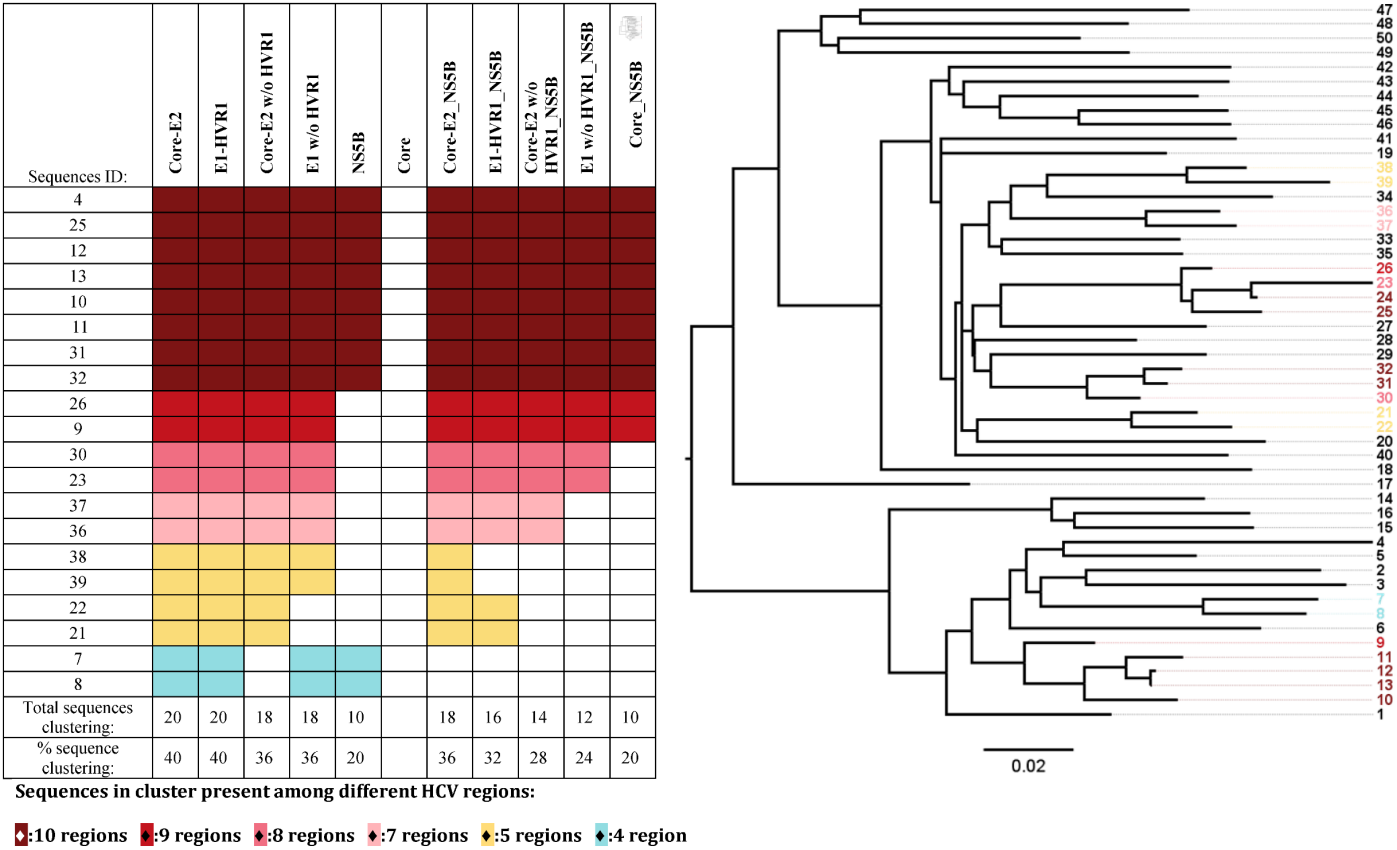
Heatmap of the number of clustered sequences present among different HCV regions using genetic distance and clustering threshold criteria and illustrated in a phylogram using Core-E2 region. Sequences with a pairwise distance below the genetic threshold were identified as part of a cluster. The colours represent the number of regions which consistently identified each sequence in a cluster. A phylogram based on the Core-E2 analysis highlights sequences that are included/excluded of cluster depending of the region selected.

Further analysis demonstrated that Bootstrapping influenced clustering membership (Fig 8A). Analysis of the Core-E2 region by Cluster Picker did not include Participant #9 (highlighted by a red star) as the threshold for this node was below 90%, while this participant was included if concatenated with NS5B. However, if a pairwise distance only method was used for clustering analysis (without any consideration of bootstrap value), this participant was included in the cluster for Core-E2 alone (Fig 9, participant 9).

#### Bootstrap threshold influences the percentage of participants clustering in more conserved regions

Fig 8B showed the sensitivity analysis with bootstrap support threshold varying between 70% and 98% for Core-E2, Core-E2_NS5B and NS5B with their respective genetic clustering threshold (top graphs) or with genetic distance threshold relaxed at 0.08 for all (bottom graphs). With specific regions’ maximum genetic distance clustering threshold, Core-E2 and Core-E2_NS5B showed a constant percentage of clustering despite bootstrap support variation. NS5B was the only region affected where clustering decreased as bootstrap support was increased to 98%. With genetic threshold relaxed at 0.08, the percentage of sequence clustering was increased for Core-E2 and Core-E2_HVR1 with bootstrap threshold below 90%.

### Clustering without bootstrap threshold using pairwise distance increases percentage clustering for most regions

Clustering for each region was also assessed using only the genetic distance distribution and the threshold define in panel i in Figs 5–7 to exclude the effect of including bootstrap threshold (Table 1, last column). Most regions demonstrated an increase in the number of clustered sequences compared to that selected by Cluster Picker with a bootstrap threshold of 90%, although regions containing HVR1 showed equivalent numbers of clustered sequences when concatenated with NS5B.

### Highly related sequences consistently cluster across most regions

Fig 9A heatmap describes the sequences included in clusters for each region as analysed using the genetic distance and the genetic distance clustering threshold set in panel i Figs 5–7 (no bootstrap threshold). Each sequence was highlighted on the tip of a phylogenetic tree based on the Core-E2 region (Fig 9B). Eight sequences were consistently included in clusters generated by all ten regions (Fig 9A, heatmap brown colour). Core was not selected due to the lack of genetic threshold for clustering. As the genetic distance increases (as shown by the branch lengths), the number of times sequences were identified as part of a cluster in each region decreased.

### Weighted Robinson-Foulds metrics to compare phylogenetic trees among HCV regions

**Fig 10:**
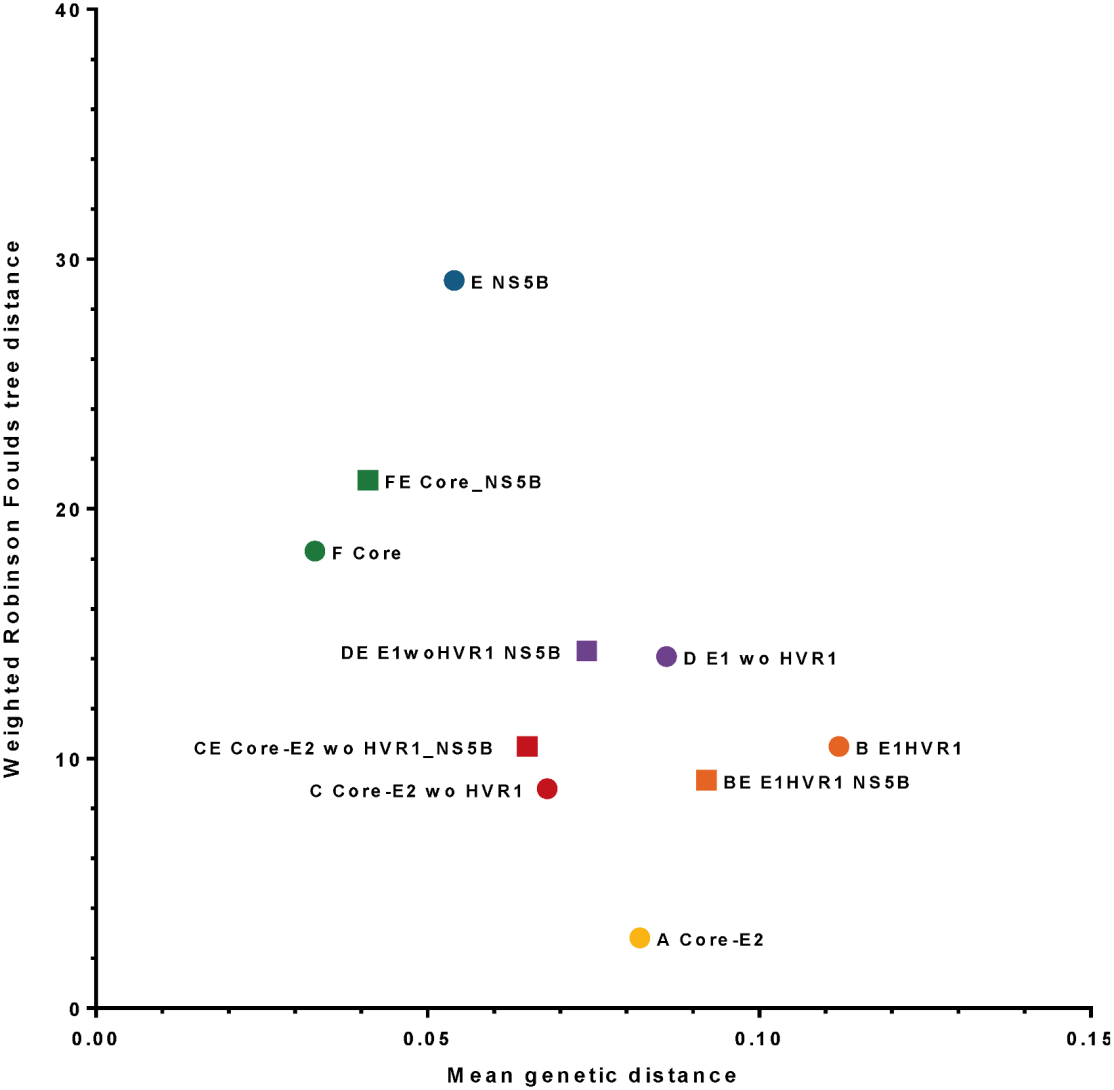
Weighted Robinson-Foulds tree distances among HCV regions compared to the Core-E2_NS5B tree have been compared to mean genetic distance of ATAHC HCV sequences. First values from weighted Robinson-Foulds tree distances computed by RAxML1.3 from HCV regions compared to the region Core-E2 concatenated to NS5B as reference with length of 1684bp and a mean genetic distance of 0.076).

The Robinson-Foulds (RF) tree topology, using Core E2 concatenated to NS5B as the reference sequence was compared with either mean genetic distance (Fig 10) or sequence length (S8 and S9 Figs). The non-concatenated regions showed the RF distance accumulation (increase of tree topology variation) from Core-E2 to Core, with NS5B demonstrating the highest RF distance.

## Discussion

Many methods exist to infer phylogenetic trees and measure statistical support [25] [26]. This study used a number of phylogenetic approaches to systematically assess the impact of HCV region on clustering analysis among people with recent HCV infection. The results demonstrated that the genetic diversity of the region, concatenation of two regions and inclusion of reference sequences all influence tree topology and phylogenetic clustering results. The pan-genotypic Core-E2 sequencing protocol described here may provide an optimised combination of diversity and length that permits a range of phylogenetic analyses. This study highlights the importance of careful consideration the selected HCV region to ensure the research questions relevant to the epidemic of each study are addressed.

The genetic diversity within a region of HCV is influenced by the mode and rate of transmission among the population affected [27] and environmental impacts including treatment [3], all of which may influence clustering analysis. This analysis demonstrated how the choice of a clustering threshold within a region influenced clustering outcome among untreated participants with recent HCV infection. A more conservative (lower) threshold is likely to increase the confidence of highly related transmission clusters, while a less conservative (higher) threshold is likely include larger clusters of less related participants. The latter might be applicable for broader population based studies. To standardise comparisons between regions, the clustering threshold for this study was determined by the overlap of the distribution curves of pairwise distance between the most-closely and distantly related sequences. Regions with greater diversity, such as Core-E2 or E1-HVR1, showed higher genetic clustering threshold and percentage clustering results than shorter, more conserved regions. Although the Core region is often used in phylogenetic analysis [28–30], this study found it was too conserved for clustering analysis. This study also found partial NS5B to be less informative than Core-E2 region, while other studies have used it for phylogenetic analyses [31–35] and suggest it is particularly suitable dating the introduction of an infection into a population using Bayesian methodologies [36–38].

Concatenating NS5B to other regions has been used for its presumed statistical advantage of greater phylogenetic accuracy by increasing sequence data for the given set of taxa [10, 39]. Concatenation of the short, conserved NS5B used in this study had limited benefit as demonstrated by the minimal impact on RF metrics. Nevertheless, NS5B did improve results when concatenated to Core and may be applied to existing sequence genotyping data in clinical cohorts [40, 41]. The analysis of Core and NS5B alone, and concatenated together, may be useful to discover potential recombination events [42], although potential infection with multiple strains of HCV would need to be ruled out. While adding reference sequences, or an out-group, are important for rooting the sequences of interest in the substitution tree model, the addition of references had limited effect in this study. However, in this context, HVR1 did increase the patristic distance due to higher divergence between local cohort and global reference sequences. The rapid divergence of HVR1 impacts genetic relatedness and may be particularly useful to analyse closely related transmission events in recent epidemic and intra-host-viral evolution studies [43–47]. The removal of HVR1, however, may be more useful for broader population clustering analyses.

Overall, these results indicate the importance of understanding the influence of the genomic region and the criteria by which clusters are defined. Two related definitions of clusters were used in this study: (i) a monophyletic group of sequences that share a common ancestor, typically with strong bootstrap or posterior probability support (as genetic distance and bootstrap threshold support estimated by Cluster Picker in this study), (ii) two or more sequences whose pairwise genetic distances fall below some threshold. The difference between these definitions is crucial for rapidly evolving sequences as the divergence through time means that lineages will eventually fail criterion (ii) whilst still meeting criterion (i). In our study, regions Core-E2, Core-E2 w/o HVR1, E1-HVR1 and E1 w/o HVR1 using method (ii) demonstrate very high clustering and probably have been overestimated compared to method (i). Furthermore, clustering is decreased when HVR1 is removed for method (i) indicating that the use of bootstrap and the removal of highly genetically diverse regions might be more adequate to infer transmission clusters. Several studies have shown that high sequence similarity could impair accurate phylogenetic trees [48, 49]. Bootstrap support alone to define clusters might not be the preferred method in our study as it shows high result discrepancies between HCV regions in tree topology with higher statistical support for longer regions such as Core-E2 than the shorter NS5B regions. Our study indicates that Core-E2 without HVR1 sequences analysed with Cluster Picker with both support criteria (bootstrap and maximum genetic distance) appears to be the best method for cluster analysis.

This study has a number of limitations. Although the Core-E2 amplicon provides more genetic information and analytical options than other regions assessed in this manuscript, it is likely to have a higher limit of detection than NS5B due to amplicon size. The potential influence of regions not analysed in this study is also not known and may be comprehensively addressed analysing full genome sequences using the approaches similar to those described here. The amplification of full length sequences from a larger dataset that include other genotypes would enable a more comprehensive comparison of each HCV region. This study compared only a few of the large number of methodologies and algorithms available to reconstruct phylogeny. The work would have also benefited from clinically proven with known history to verify the inferred phylogenetic trees are, in fact, “true”. Additional within-host evolutionary data would also facilitate a more refined sensitivity analysis to help define the upper and lower boundaries of suitable genetic distance clustering threshold for each region, rather than relying on between-host pairwise distances as in this study. The analysis has been limited to one HCV genotype and the participants of the ATAHC study, recruited in Sydney and Melbourne, Australia. Future analyses with larger cohorts representing a range of epidemics, genotypes and disease stages would be needed to further validate these analyses on tree topology and phylogenetic clustering.

In summary, we have systematically demonstrated the influence of HCV region on inferred transmission clusters and that adding reference sequences, sequence concatenation and the removal of highly variable regions can influence cluster characterisation. We developed a cost-effective, pan-genotypic Core-E2 sequencing protocol that provides an optimised combination of diversity and length, suitable for many epidemiological studies. We found the use of a combination of bootstrap and genetic distance threshold to be preferable over either bootstrap support alone or pairwise distance. The selection of HCV region and phylogenetic methods require careful consideration to ensure the goals of each study are addressed.

## Supporting Information

S1 Fig Amplification for CORE-E2 HCV region altering reaction conditions for the (A) Reverse transcription (B) PCR. (DOCX)

S2 Fig Effect of PolyMate on Sanger Sequencing reaction of CORE-E2 amplicon. (DOCX)

S3 Fig Clustering results among 50 GT1a ATAHC sequences with genetic distance, percentage of sequences, tree, patristic distance and bootstrap values. (DOCX)

S4 Fig Clustering results among 50 GT1a ATAHC sequences with genetic distance, percentage of sequences, tree, patristic distance and bootstrap values. (DOCX)

S5 Fig Clustering results among 50 GT1a ATAHC sequences with genetic distance, percentage of sequences, tree, patristic distance and bootstrap values. (DOCX)

S6 Fig Patristic distance among 50 GT1a ATAHC sequences. (DOCX)

S7 Fig Patristic distance among 50 GT1a ATAHC sequences. (DOCX)

S8 Fig Mean genetic distance among HCV regions used for clustering analysis. (DOCX)

S9 Fig Weighted Robinson-Foulds tree distances among HCV regions compared to the Core-E2_NS5B tree versus length of HCV sequences used in this study. (DOCX)

S1 File (DOCX)

S1 Table Primers used for the amplification of HCV region CORE-HVR1 and NS5B. (DOCX)
