# Supplementary material for: The Influence of Hepatitis C Virus Genetic Region on Phylogenetic Clustering Analysis
Source: PLoS One. 2015 Jul 20;10(7):e0131437. doi: 10.1371/journal.pone.0131437 (PMC4507989; doi:10.1371/journal.pone.0131437)
Supplement: S1 Fig — (DOCX) [file pone.0131437.s001.docx]

**A**


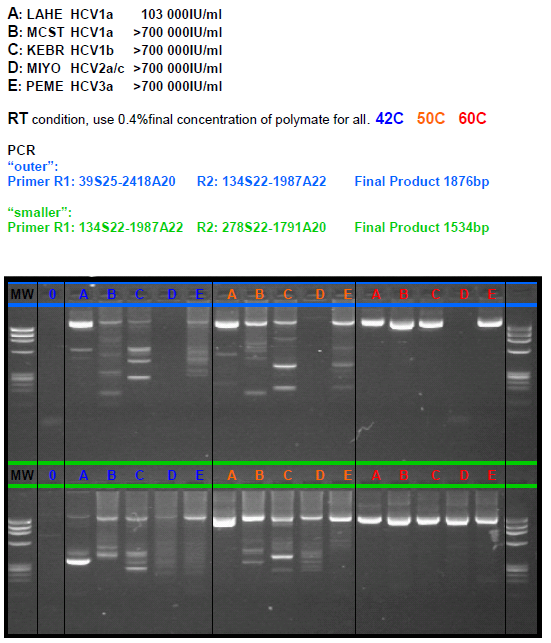


RT 42C

RT 50C

RT 60C

**Gt1a**

**Gt1a**

**Gt1b**

**Gt2a/c**

**Gt3a**

**Gt1a**

**Gt1a**

**Gt1b**

**Gt2a/c**

**Gt3a**

**Gt1a**

**Gt1a**

**Gt1b**

**Gt2a/c**

**Gt3a**

**B**


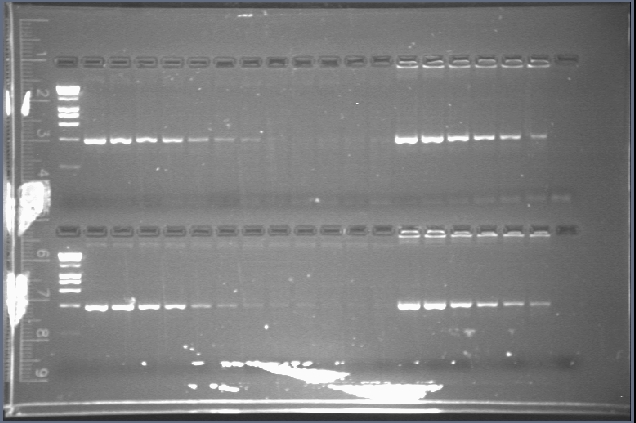

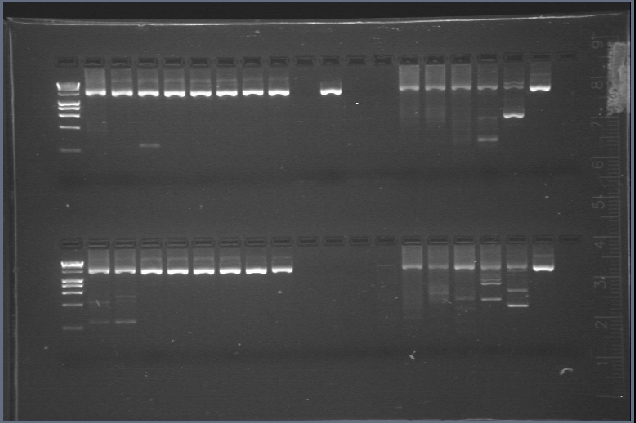


6.5

6

5.5

5

4.5

4

3.5

3

2.5

2

1.5

NEG

6

5.5

5

4.5

4

3.5

NEG

Log viral load, HCV Gt1a

CORE-E2

RT 60ºC RT 42ºC

+ polymate - polymate

NS5B

**S1 Figure: Amplification for CORE-E2 HCV region altering reaction conditions for the (A) Reverse transcription (B) PCR**
