# Supplementary material for: The Influence of Hepatitis C Virus Genetic Region on Phylogenetic Clustering Analysis
Source: PLoS One. 2015 Jul 20;10(7):e0131437. doi: 10.1371/journal.pone.0131437 (PMC4507989; doi:10.1371/journal.pone.0131437)
Supplement: S2 Fig — (DOCX) [file pone.0131437.s002.docx]

.


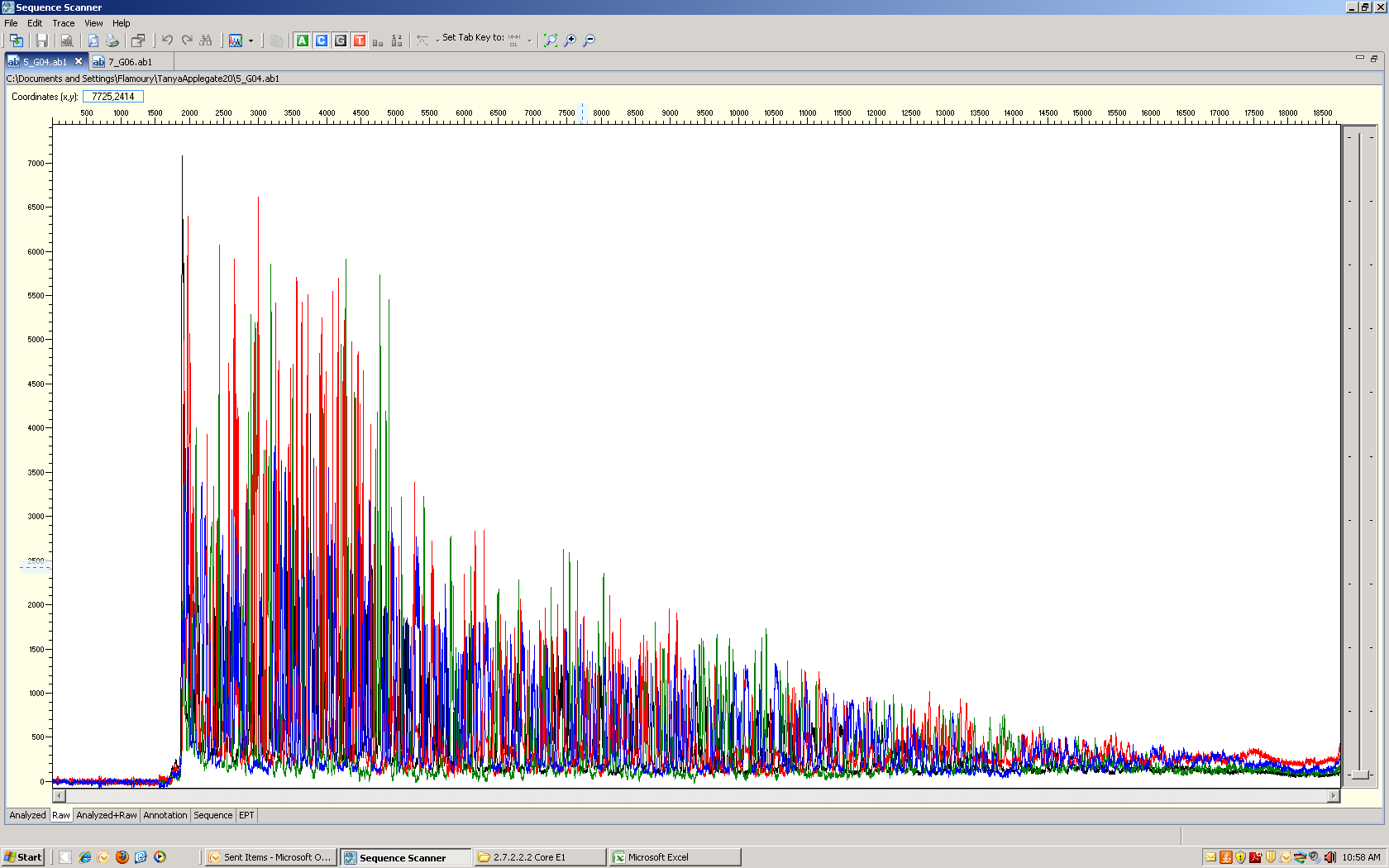

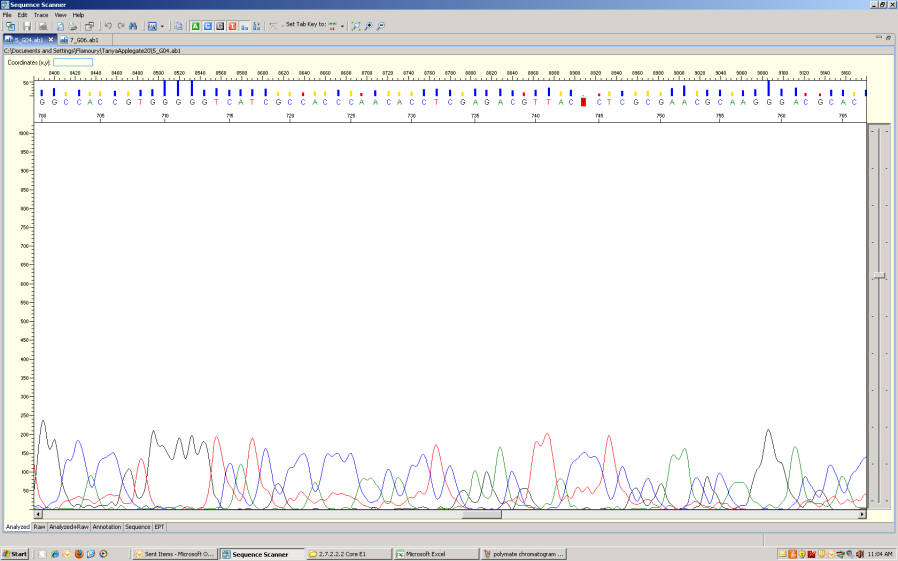

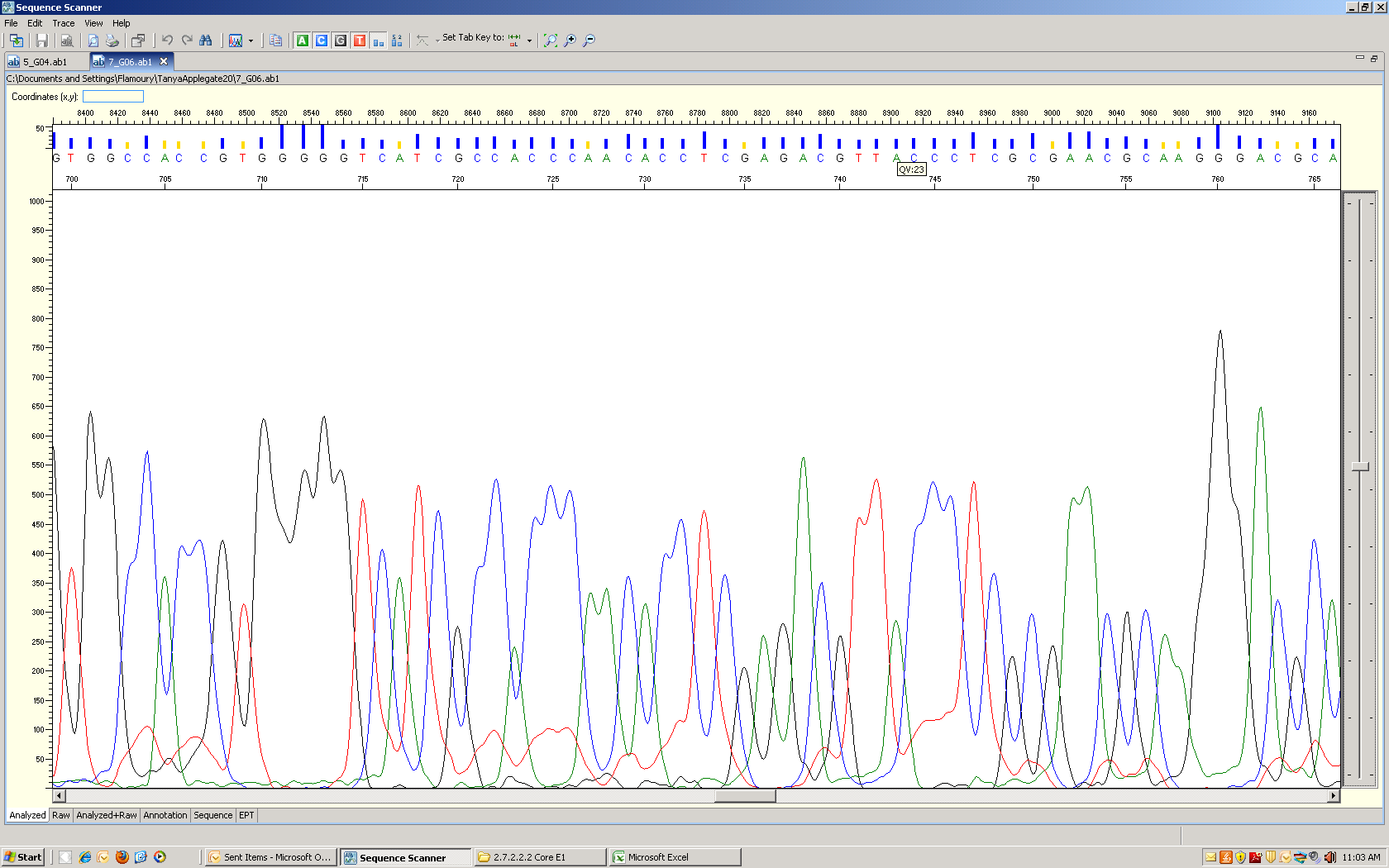


Raw fluorescence

Analyzed fluorescence on Chromatogram

Without PolyMate


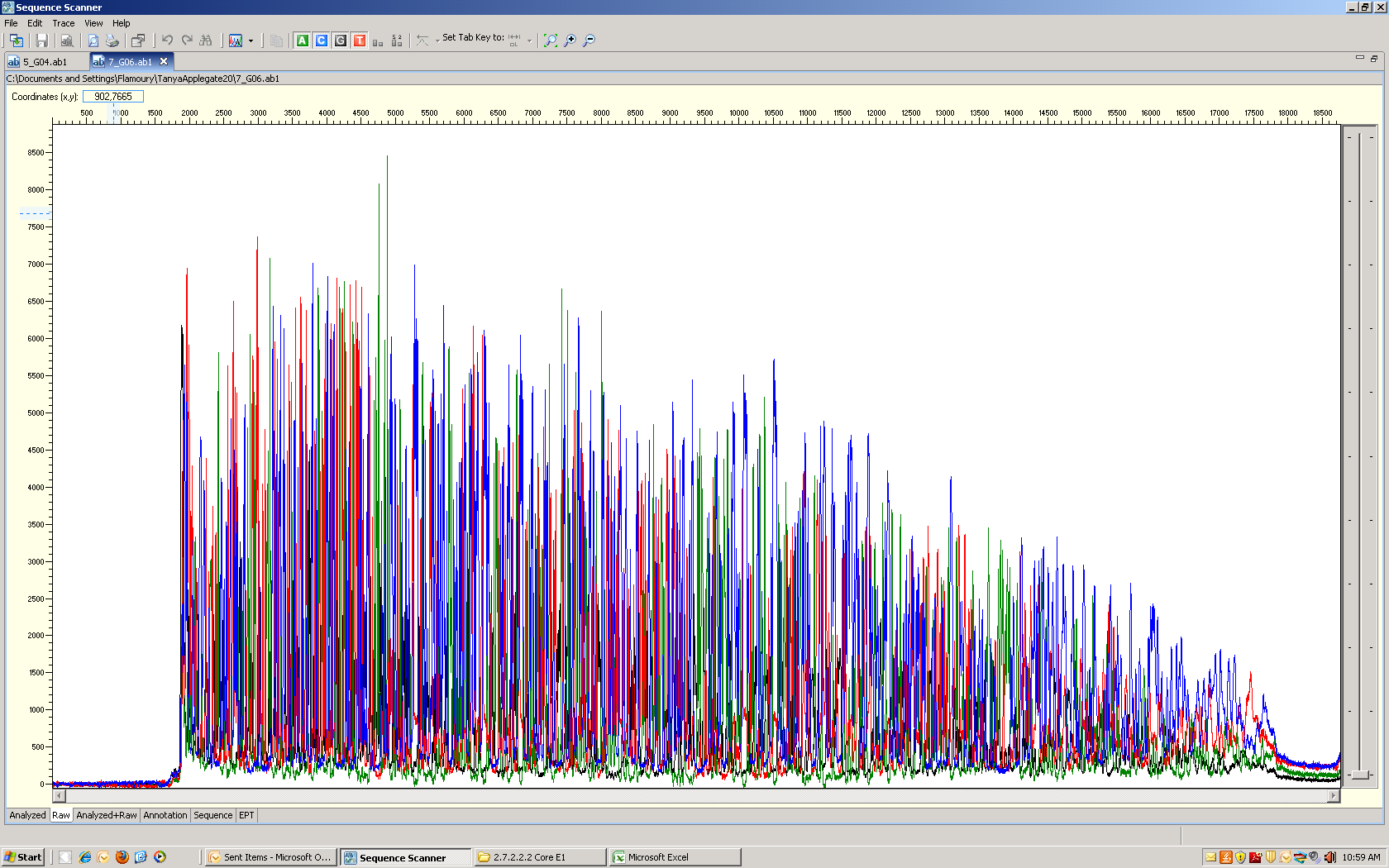


With PolyMate

**S2 Figure: Effect of PolyMate on Sanger Sequencing reaction of CORE-E2 amplicon.**
