# Supplementary material for: The Influence of Hepatitis C Virus Genetic Region on Phylogenetic Clustering Analysis
Source: PLoS One. 2015 Jul 20;10(7):e0131437. doi: 10.1371/journal.pone.0131437 (PMC4507989; doi:10.1371/journal.pone.0131437)
Supplement: S3 Fig — (DOCX) [file pone.0131437.s003.docx]

**Core-E2_NS5B**

**i**

Genetic distance

Genetic distance

**Core-E2 w/o HVR1_NS5B**

**ii**

***
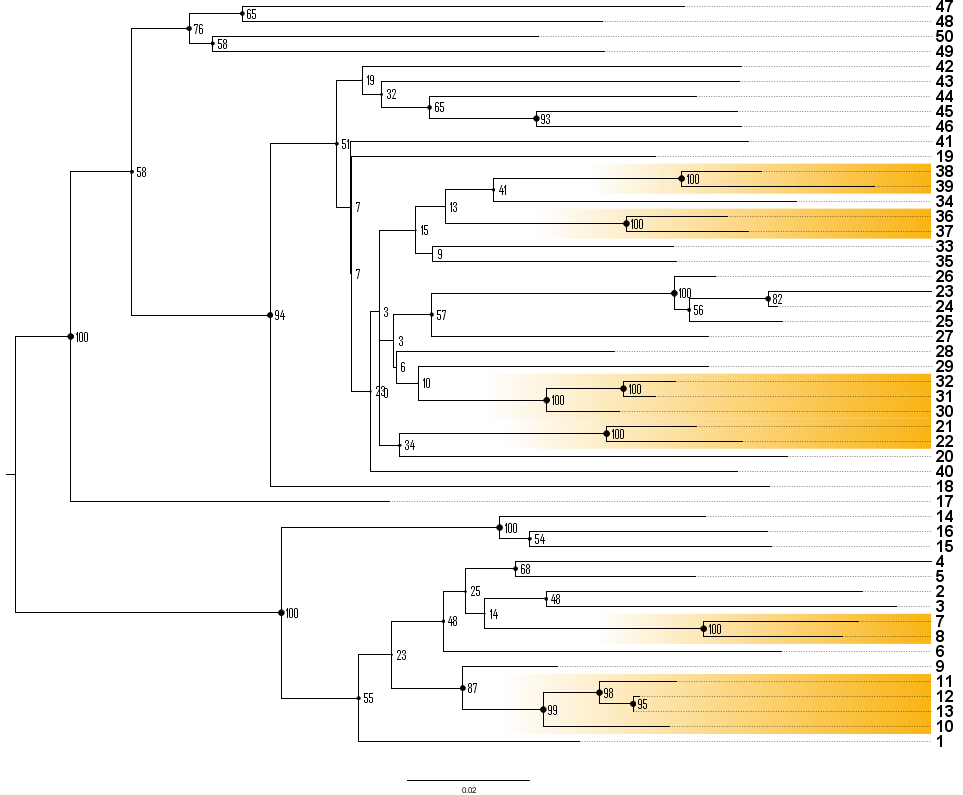
***

Percentage of sequence clustering and average cluster size

Percentage of sequence clustering and average cluster size

Phylogenetic tree

**iii**

Phylogenetic tree

**
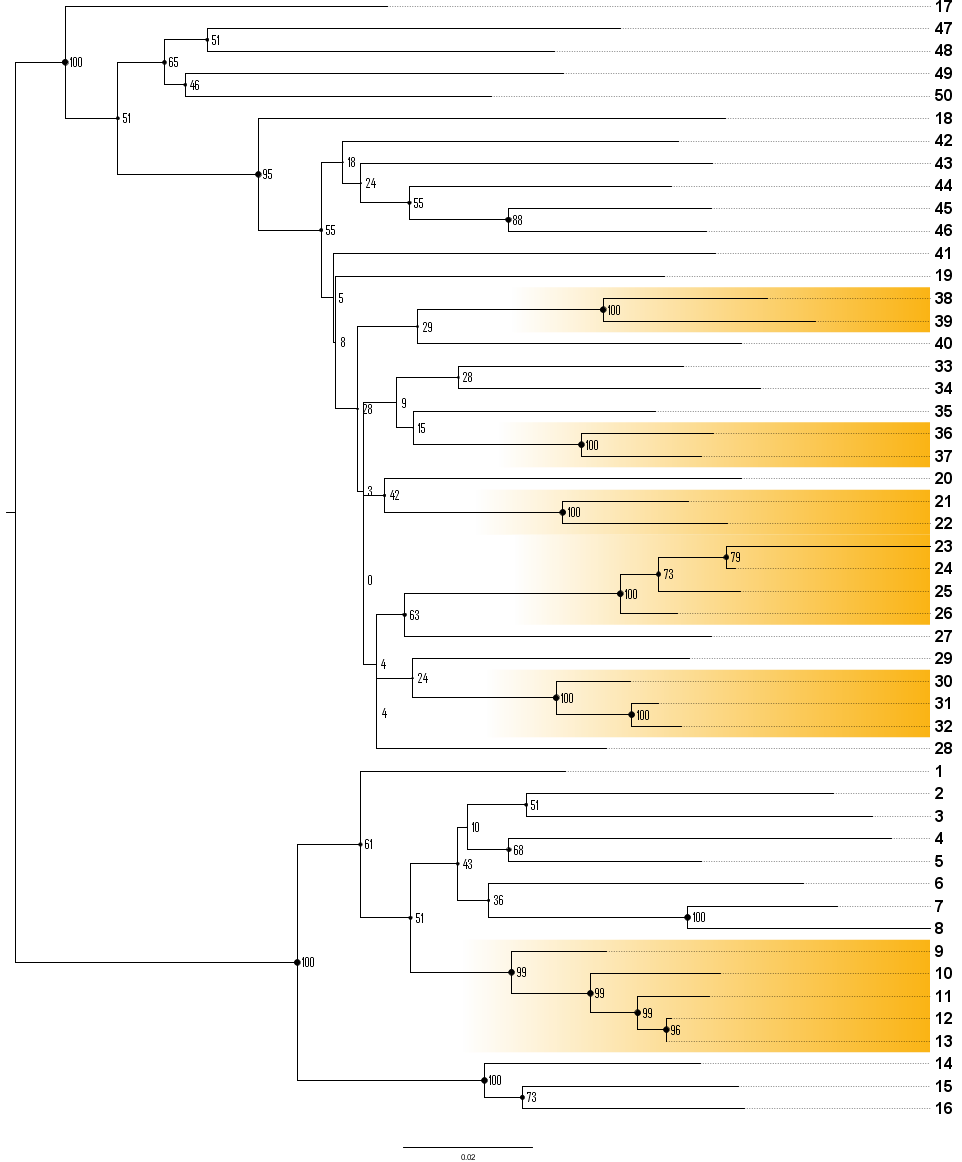

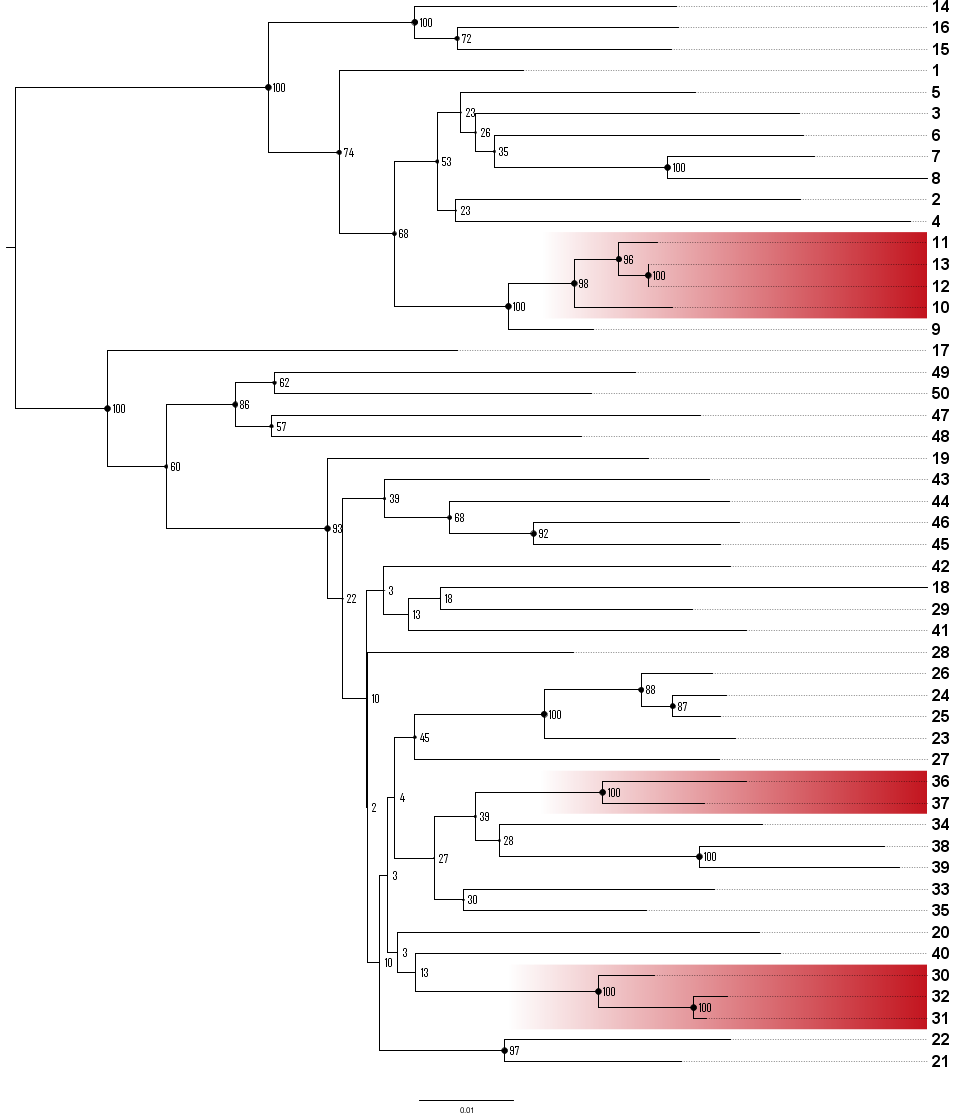
**

Genetic distance

A

v

e

r

a

g

e

c

l

u

s

t

e

r

s

P

a

t

r

i

s

t

i

c

d

i

s

t

a

n

c

e

A

v

e

r

a

g

e

b

o

o

t

s

t

r

a

p

(

d

a

s

h

e

d

)

**0.00**

**0.01**

**0.02**

**0.03**

**0.04**

**0.05**

**0.06**

**0.07**

**0.08**

**0.09**

**0.00**

**0.02**

**0.04**

**0.06**

**0.08**

**0.10**

**0.12**

**90**

**92**

**94**

**96**

**98**

**100**

ATAHC

ATAHC+LANL

**S3 Figure: Clustering results among 50 GT1a ATAHC sequences with genetic distance, percentage of sequences, tree, patristic distance and bootstrap values.**
