# Supplementary material for: The Influence of Hepatitis C Virus Genetic Region on Phylogenetic Clustering Analysis
Source: PLoS One. 2015 Jul 20;10(7):e0131437. doi: 10.1371/journal.pone.0131437 (PMC4507989; doi:10.1371/journal.pone.0131437)
Supplement: S4 Fig — (DOCX) [file pone.0131437.s004.docx]

Genetic distance

**E1 w/o HVR1_NS5B**

**E1-HVR1_NS5B**

Genetic distance

**i**

Percentage of sequence clustering and average cluster size

Percentage of sequence clustering and average cluster size

**ii**

**iii**

Phylogenetic tree

Phylogenetic tree

***
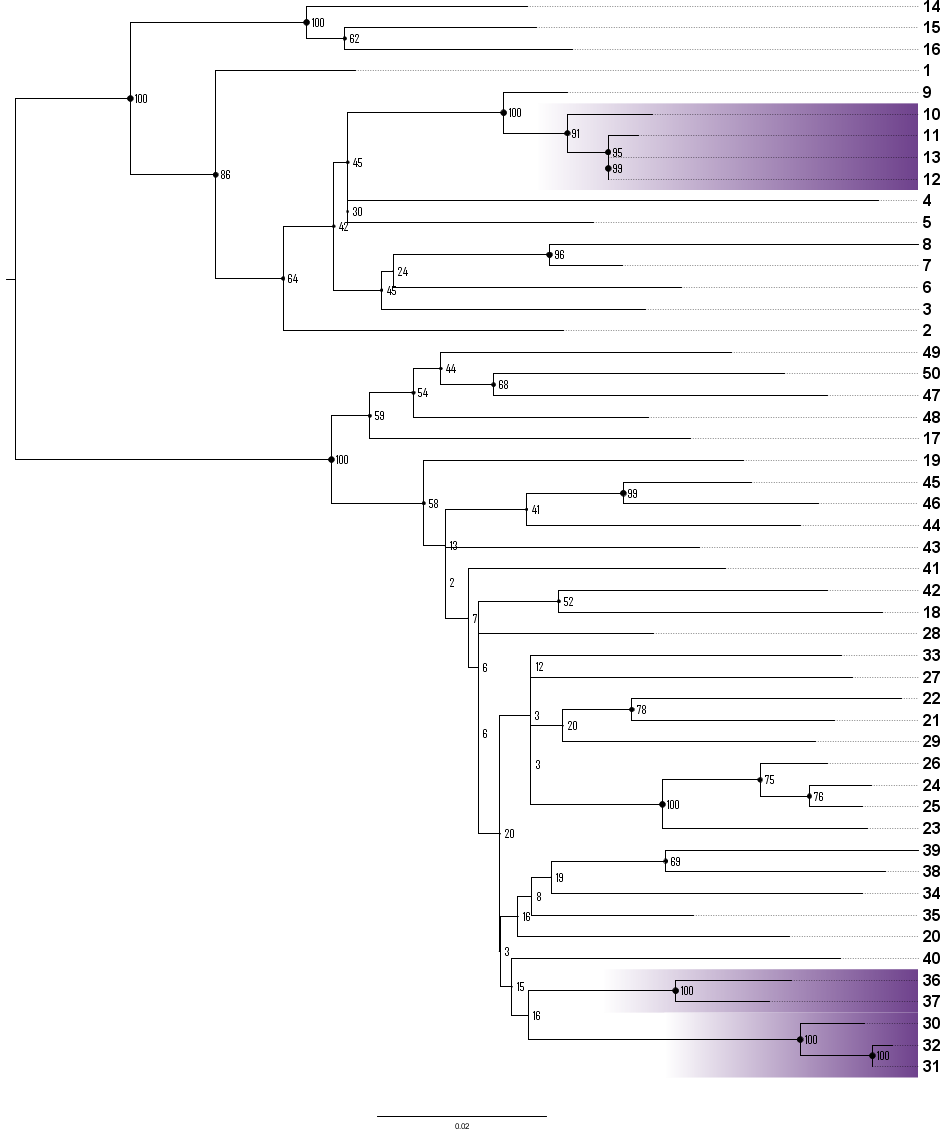
*
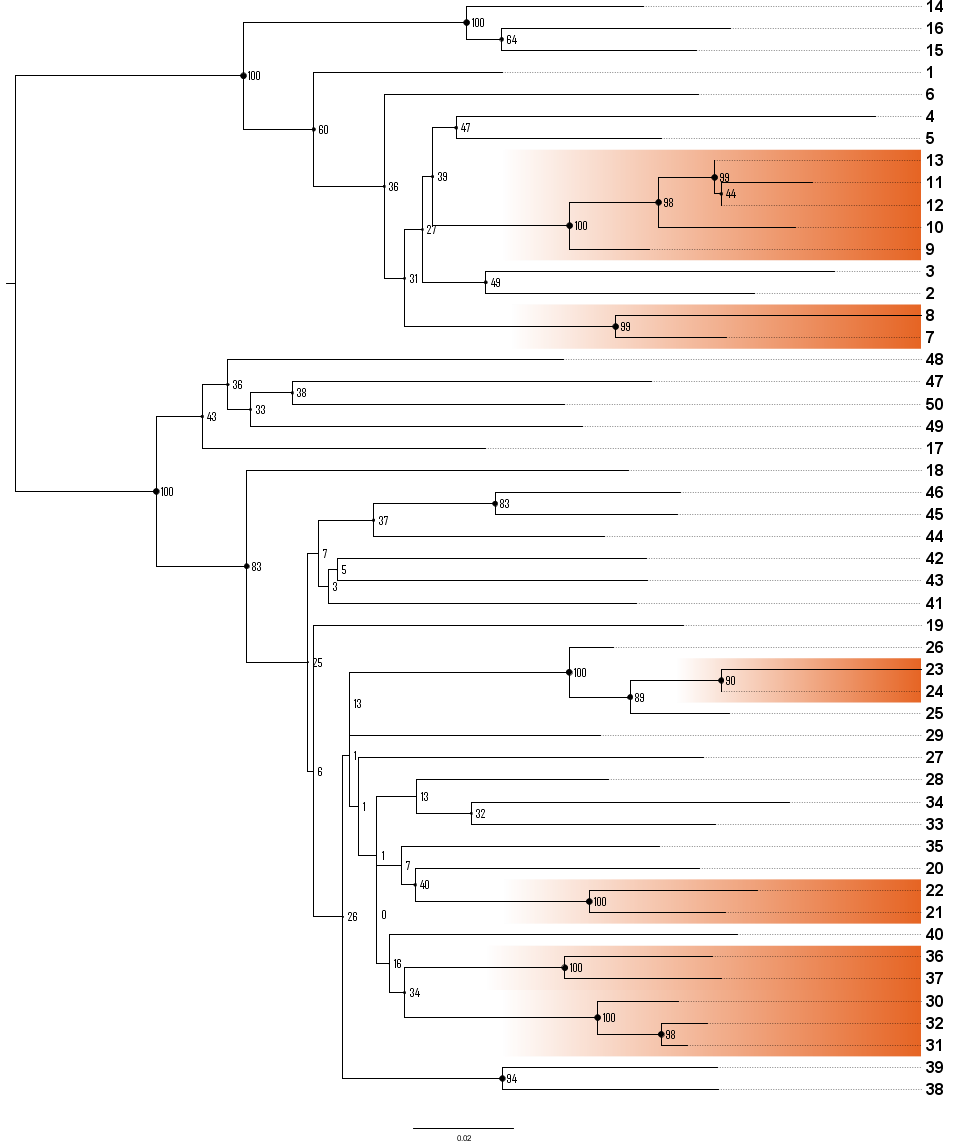
**

**S4 Figure: Clustering results among 50 GT1a ATAHC sequences with genetic distance, percentage of sequences, tree, patristic distance and bootstrap values.**
