# Supplementary material for: The Influence of Hepatitis C Virus Genetic Region on Phylogenetic Clustering Analysis
Source: PLoS One. 2015 Jul 20;10(7):e0131437. doi: 10.1371/journal.pone.0131437 (PMC4507989; doi:10.1371/journal.pone.0131437)
Supplement: S6 Fig — (DOCX) [file pone.0131437.s006.docx]

**Core-E2 w/o HVR1**

**Core-E2**

**E1 w/o HVR1**

**E1-HVR1**

**CORE**

**NS5B**

**S6 Figure: Patristic distance among 50 GT1a ATAHC sequences.**
