# Supplementary material for: The Influence of Hepatitis C Virus Genetic Region on Phylogenetic Clustering Analysis
Source: PLoS One. 2015 Jul 20;10(7):e0131437. doi: 10.1371/journal.pone.0131437 (PMC4507989; doi:10.1371/journal.pone.0131437)
Supplement: S7 Fig — (DOCX) [file pone.0131437.s007.docx]

**Core-E2 w/o HVR1_NS5B**

**Core-E2_NS5B**

**E1-HVR1_NS5B**

**E1 w/o HVR1_NS5B**

**CORE_NS5B**

**S7 Figure: Patristic distance among 50 GT1a ATAHC sequences.**
