# Supplementary material for: The Influence of Hepatitis C Virus Genetic Region on Phylogenetic Clustering Analysis
Source: PLoS One. 2015 Jul 20;10(7):e0131437. doi: 10.1371/journal.pone.0131437 (PMC4507989; doi:10.1371/journal.pone.0131437)
Supplement: S9 Fig — (DOCX) [file pone.0131437.s009.docx]

**S9 Figure: Weighted Robinson-Foulds tree distances among HCV regions compared to the Core-E2_NS5B tree versus length of HCV sequences used in this study.**
