# Supplementary material for: The Influence of Hepatitis C Virus Genetic Region on Phylogenetic Clustering Analysis
Source: PLoS One. 2015 Jul 20;10(7):e0131437. doi: 10.1371/journal.pone.0131437 (PMC4507989; doi:10.1371/journal.pone.0131437)
Supplement: S1 File — (DOCX) [file pone.0131437.s010.docx]

# Supporting Information

**S1 Fig. Amplification for Core-E2 HCV region altering reaction conditions for the (A) Reverse transcription (B) PCR**

(A) Viral RNA was reverse transcribed (RT) with the VILO system (Invitrogen) using Superscript III and random hexamers and PCR additive PolyMate (Bioline) at three different temperatures for one hour RT: 42°C, 50°C and 60°C. (B) Limit of detection of PCR amplification of Core-HVR1 (1000IU/mL) and NS5B (3000IU/mL) of serial dilutions of known positive plasma samples as shown on agarose gel of PCR products after PCR amplification. The reverse transcription reaction was performed with both polymate at 60ºC and without at 42ºC for comparison

**S2 Fig. Effect of PolyMate on Sanger Sequencing reaction of Core-E2 amplicon.**

Polymate used for sequencing reaction removed secondary structures as shown on the Core-HVR1 sequencing raw fluorescence graphs (top), which improved the length of the sequence and sCore quality for each nucleotide (higher pic fluorescence, bottom graphs).

**S3 S4 and S5 Figs. Clustering results among 50 GT1a ATAHC sequences with genetic distance, percentage of sequences, tree, patristic distance and bootstrap values.**

**S6 and S7 Figs. Patristic distance among 50 GT1a ATAHC sequences**

Average patristic distances (plain lines) and bootstrap support (dot lines) using PATRISTIC (patristic distance) and Cluster Picker (bootstrap support) with maximum genetic distance threshold varied between 0.01 and 0.08. Colour lines represent ATAHC sequences only and grey lines additional LANL reference sequences. The vertical dot line represents the clustering threshold illustrated in Fig. 5 panel i and Supplementary Fig. 3 panel i to determine the average cluster patristic distance and average cluster bootstrap support (Table 1).

**S8 Fig. Mean genetic distance among HCV regions used for clustering analysis.**

**S9 Fig. Weighted Robinson-Foulds tree distances among HCV regions compared to the Core-E2_NS5B tree versus length of HCV sequences used in this study.**

**S1 Table. Primers used for the amplification of HCV region Core-HVR1 and NS5B.**

The forward primers were designed using 280 sequences (116 1a, 105 1b, 19 2a, 21 3a and 19 6a) from the 5’UTR region to middle of E1 (H77 location 100 to 1200bp) with a focus on the conserved region of 5’UTR and early Core. The reverse primers have been designed using 1772 sequences (697 1a, 465 1b, 3 1c, 20 2a, 27 2b, 1 2c, 1 2i, 1 2k, 1217 3a, 1 3b, 1 3k, 14 4a, 1 5a, 15 6a, 1 6b, 2 6d, 3 6f and 2 6q) from the E2 gene (H77 location 1491 to 2580bp).

## Supplementary material

### Core-E2 assay development and characterisation.

To improve the efficiency of the transcription and amplification of HCV, Polymate and a range of conditions were evaluated for this protocol (Figure A in S1 Fig.). Among the conditions assessed, including magnesium titrations, temperature gradient and “touch-down” and PCRx enhancers, the use of Polymate during the Reverse transcriptase at a temperature of 60ºC was critical for successful subsequent amplification of the Core-E2 amplicon of most HCV genotypes for both regions. The limits of detection of the Core-E2 and NS5B were 1000IU/mL and 3000IU/mL respectively (Figure B in S1 Fig.).

### Design and development of Core-HVR1

Forward primers for the amplification of were designed in-house using HCV sequences from LANL. The forward primers for the Core-E2 were designed using 280 sequences (116 1a, 105 1b, 19 2a, 21 3a and 19 6a) from the 5’UTR region to middle of E1 (H77 location 100 to 1200bp) with a focus on the conserved region of 5’UTR and early Core. The reverse primers have been designed using 1772 sequences (697 1a, 465 1b, 3 1c, 20 2a, 27 2b, 1 2c, 1 2i, 1 2k, 1217 3a, 1 3b, 1 3k, 14 4a, 1 5a, 15 6a, 1 6b, 2 6d, 3 6f and 2 6q) from the E2 gene (H77 location 1491 to 2580bp). Three pairs of primers were designed with forward primers located in the 5’UTR region with H77 HCV location at 39 to 63bp, 134 to 155bp and 278 to 299bp. Reverse primers contained between 4 and 5 degenerative bases to be able to identify most genotypes sequences with their location in E2: 1791 to 1810bp, 1987 to 2008bp and 2418 to 2437bp. After reverse transcription of the viral RNA with the Vilo system and random hexamers at 42°C for one hour, amplification was performed by nested PCR using Platinum Taq DNA Polymerase (Life Technologies) hot-start enzyme with a final magnesium chloride concentration of 1.5mM. Several ex-diagnostic plasma samples with high viral load from different HCV genotypes were used and only a single Gt1a was amplified with the 2 pairs of primers listed in the table 1 (data not shown). To improve the success of the transcription and amplification of HCV, Polymate and conditions were evaluated for this protocol (Fig. 1A). Other conditions were assessed such as magnesium, temperature gradient and “touch-down” for PCR and other additive such as PCRx enhancer system from Invitrogen. As shown in figure A in S1 Fig., viral RNA was reverse transcribed (RT) with the VILO system (Invitrogen) and PCR additive PolyMate (Bioline) for one hour at three different temperatures: 42°C, 50°C and 60°C. The RT at 60°C with Polymate allowed subsequent amplification of all HCV genotypes tested. Use of Polymate for the Reverse transcriptase at a temperature of 60ºC was critical for successful subsequent amplification of the Core-HVR1amplicon of most HCV genotypes. High fidelity Velocity DNA polymerase (Bioline) was subsequently used to minimize PCR errors with the same conditions. The reverse transcription reaction was performed with both polymate at 60ºC and without at 42ºC for comparison. In the latter condition, the amplification of the Core-E2 was problematic nevertheless NS5B could be amplified with both RT conditions.

### Generation of Core-E2 and NS5B amplicon

NS5B (388bp) was amplified by a single round PCR as previously described [6] with some modifications to reaction conditions (see Supplementary Material for details). The Core-E2 region (1534bp) was amplified by an in-house nested PCR assay. Forward and reverse consensus primers for Core-E2 were designed to amplify most genotypes using 280 and 1772 Los Alamos HCV Sequence database (LANL) sequences respectively (S1 Table). Final PCR reactions contained 1× HI-FI buffer, 0.02 unit/µL of Velocity Taq Polymerase (Bioline), 250 µM dNTP (Bioline), 200 nM forward primer (Integrated DNA Technologies), 400 nM reverse primer, one times concentration of PolyMate (Bioline) and cDNA at 1/10 of the final reaction volume. Round 1 PCRs for Core-HVR1 and NS5B were performed in a total volume of 20 µL, round 2 PCRs for Core-HVR1 in 40 µL and NS5B repeats in 50 µL total reactions. Thermocycling conditions were as follows; first round PCR was carried out for 98°C for 2 min, then 35 cycles of 98°C, 60°C and 72°C for 30 sec, 30 sec and 1 min respectively and followed by 72°C for 10 min; second round PCR for the Core-E2 fragment or the single round NS5B are as described for Core-E2, with the exception of requiring 40 cycles and an annealing temperature at 56°C. PCR amplicon size and quality was confirmed by 2.5% agarose gel electrophoresis.

### Sequencing reactions

PCR product was either purified directly using the QIAquick PCR purification kit spin column (Qiagen) or by QIAquick Gel extraction kit spin column (Qiagen) after electrophoresis to remove non-specific amplicons as required. Sanger sequencing of PCR products in both directions was completed by the Australian Genome Research Facility Ltd (Westmead, NSW, Australia) using ABI Prism BigDye terminator chemistry (BDT v3.1, Applied Biosystem, USA), using KB Basecaller and Sequence Scanner v1.0 software (Applied Biosystem). Sequencing reactions contained 6µL template (purified or 1/15 diluted PCR product for single NS5B fragment), 5µL PolyMate and 1µL primer at 20µM. Raw sequences were aligned and HCV location compared to the H77 genotype 1a sequence (GenBank accession number: NC004102).

### Sequencing of Core-E2

PCR additives such as Polymate were used for sequencing reaction to remove secondary structures, as shown in S2 Fig. (upper panels) in the Core-E2 sequencing raw fluorescence. The polymerase was presumably unable to progress through some stem-loop DNA forms as shown by the typical “step” or sharp drop of fluorescence intensity on the graph of raw data. The same amplicon used for sequencing reaction with Polymate (1X final concentration) had the secondary structure removed which greatly improved the length of the sequence and also the quality score for each nucleotide (S2 Fig., Lower panels).

### Discussion on Core-E2 protocol

The Core-E2 fragment described in this study incorporated several design features. Random hexamers were used to allow subsequent amplification of any HCV region for other applications, such as mixed infection assays, to maximise applications of limited clinical sample. They also limit primer bias and help overcome the large genetic diversity within the HCV genus. Universal primers were designed to amplify the most prevalent genotypes within our cohort to reduce cost and increase throughput. The Core-E2 assay incorporated a PCR-additive, Polymate, during reverse transcription, amplification and sequencing reactions to destabilise the 3’ stem-loop secondary structures [1] and improve the sequencing success rate. The Core-E2 assay was compared with a published NS5B protocol, which demonstrated a higher sequencing success rate (90% vs 74%), probably due to the more conserved region and shorter amplicon size [7] [8]. This research assay provides several advantages over whole genome methods [5] [2] [3] for which the majority of sequence might not be used for phylogenetic clustering analysis, including amplification of multiple genotypes, low limit of detection [5, 4]. The full genome is more expensive due to the multiple sequencing reactions needed and more technically challenging due to long reaction times.

**References**

1 Smith DB, Simmonds P. Characteristics of nucleotide substitution in the hepatitis C virus genome: constraints on sequence change in coding regions at both ends of the genome. Journal of molecular evolution. 1997;45(3):238-46. Epub 1997/09/26. PubMed PMID: 9302317.

2 Lu L, Li C, Yuan J, Lu T, Okamoto H, Murphy DG. Full-length genome sequences of five hepatitis C virus isolates representing subtypes 3g, 3h, 3i and 3k, and a unique genotype 3 variant. The Journal of general virology. 2013;94(Pt 3):543-8. Epub 2012/11/16. doi: 10.1099/vir.0.049668-0. PubMed PMID: 23152370; PubMed Central PMCID: PMC3709609.

3 Li C, Lu L, Zhang X, Murphy D. Entire genome sequences of two new HCV subtypes, 6r and 6s, and characterization of unique HVR1 variation patterns within genotype 6. Journal of viral hepatitis. 2009;16(6):406-17. Epub 2009/03/14. doi: 10.1111/j.1365-2893.2009.01086.x. PubMed PMID: 19281488.

4 Grebely J, Pham ST, Matthews GV, Petoumenos K, Bull RA, Yeung B, et al. Hepatitis C virus reinfection and superinfection among treated and untreated participants with recent infection. Hepatology. 2012;55(4):1058-69. Epub 2011/10/28. doi: 10.1002/hep.24754. PubMed PMID: 22031335; PubMed Central PMCID: PMC3310282.

5 Zhang EZ, Bartels DJ, Frantz JD, Seepersaud S, Lippke JA, Shames B, et al. Development of a sensitive RT-PCR method for amplifying and sequencing near full-length HCV genotype 1 RNA from patient samples. Virology journal. 2013;10:53. Epub 2013/02/14. doi: 10.1186/1743-422X-10-53. PubMed PMID: 23402332; PubMed Central PMCID: PMC3575352.

6 Murphy DG, Willems B, Deschenes M, Hilzenrat N, Mousseau R, Sabbah S. Use of sequence analysis of the NS5B region for routine genotyping of hepatitis C virus with reference to C/E1 and 5' untranslated region sequences. Journal of clinical microbiology. 2007;45(4):1102-12. Epub 2007/02/09. doi: 10.1128/JCM.02366-06. PubMed PMID: 17287328; PubMed Central PMCID: PMC1865836.

7. Cai Q, Zhao Z, Liu Y, Shao X, Gao Z. Comparison of three different HCV genotyping methods: core, NS5B sequence analysis and line probe assay. International journal of molecular medicine. 2013;31(2):347-52. Epub 2012/12/18. doi: 10.3892/ijmm.2012.1209. PubMed PMID: 23241873.

8. Avo AP, Agua-Doce I, Andrade A, Padua E. Hepatitis C virus subtyping based on sequencing of the C/E1 and NS5B genomic regions in comparison to a commercially available line probe assay. Journal of medical virology. 2013;85(5):815-22. Epub 2013/03/20. doi: 10.1002/jmv.23545. PubMed PMID: 23508907.

**S1 Table: Primers used for the amplification of HCV region Core-HVR1 and NS5B.** Footnotes ^1^DM101 [7], ^2^DM100 [7]

| **Direction (reaction)** | **Region** | **Name** | **Reference position (H77)** | **Sequence** |
| --- | --- | --- | --- | --- |
| Forward (1) | Core-HVR1 | HCVuniv134S22 | 134→155 | 5' AGA GCC ATA GTG GTC TGC GGA A ’3 |
| Reverse (1) | Core-HVR1 | HCVuniv1987A22 | 1987←2008 | 5’ TTC ATC CAB GTR CAR CCR AAC C ‘3 |
| Forward (2) | Core-HVR1 | HCVuniv278S22 | 278→299 | 5' GCC TTG TGG TAC TGC CTG ATA G’3 |
| Reverse (2) | Core-HVR1 | HCVuniv1791A20 | 1791←1811 | 5’ GSG TAR TGC CAG CAR TAN GG ‘3 |
| Forward (1) | NS5B | HCVuniv8250S26^1^ | 8250→8275 | 5’ TTCTCRTATGAYACCCGCTGYTTTGA ‘3 |
| Reverse (1) | NS5B | HCVuniv8616A23^2^ | 8616←8638 | 5’ TACCTVGTCATAGCCTCCGTGAA ‘3 |
|  | | | | |
