# Supplementary material for: The Influence of Hepatitis C Virus Genetic Region on Phylogenetic Clustering Analysis
Source: PLoS One. 2015 Jul 20;10(7):e0131437. doi: 10.1371/journal.pone.0131437 (PMC4507989; doi:10.1371/journal.pone.0131437)
Supplement: S1 Table — (DOCX) [file pone.0131437.s011.docx]

**S1 Table: Primers used for the amplification of HCV region CORE-HVR1 and NS5B.** Footnotes ^1^DM101 [7], ^2^DM100 [7]

| **Direction (reaction)** | **Region** | **Name** | **Reference position (H77)** | **Sequence** |
| --- | --- | --- | --- | --- |
| Forward (1) | CORE-HVR1 | HCVuniv134S22 | 134→155 | 5' AGA GCC ATA GTG GTC TGC GGA A ’3 |
| Reverse (1) | CORE-HVR1 | HCVuniv1987A22 | 1987←2008 | 5’ TTC ATC CAB GTR CAR CCR AAC C ‘3 |
| Forward (2) | CORE-HVR1 | HCVuniv278S22 | 278→299 | 5' GCC TTG TGG TAC TGC CTG ATA G’3 |
| Reverse (2) | CORE-HVR1 | HCVuniv1791A20 | 1791←1811 | 5’ GSG TAR TGC CAG CAR TAN GG ‘3 |
| Forward (1) | NS5B | HCVuniv8250S26^1^ | 8250→8275 | 5’ TTCTCRTATGAYACCCGCTGYTTTGA ‘3 |
| Reverse (1) | NS5B | HCVuniv8616A23^2^ | 8616←8638 | 5’ TACCTVGTCATAGCCTCCGTGAA ‘3 |
|  | | | | |
